# New Reassortant Clade 2.3.4.4b Avian Influenza A(H5N6) Virus in Wild Birds, South Korea, 2017–18

**DOI:** 10.3201/eid2410.180461

**Published:** 2018-10

**Authors:** Jung-Hoon Kwon, Sol Jeong, Dong-Hun Lee, David E. Swayne, Yu-jin Kim, Sun-hak Lee, Jin-Yong Noh, Tseren-Ochir Erdene-Ochir, Jei-Hyun Jeong, Chang-Seon Song

**Affiliations:** Konkuk University, Seoul, South Korea (J.-H. Kwon, S. Jeong, Y.-J. Kim, S.-H. Lee, J.-Y. Noh, T.-O. Erdene-Ochir, J.-H. Jeong, C.-S. Song);; US Department of Agriculture, Athens, Georgia, USA (D.-H. Lee, D.E. Swayne)

**Keywords:** Highly pathogenic avian influenza virus, H5N6, wild birds, South Korea, reassortant, viruses, respiratory infections, zoonoses, influenza

## Abstract

We isolated new reassortant avian influenza A(H5N6) viruses from feces of wild waterfowl in South Korea during 2017–18. Phylogenetic analysis suggested that reassortment occurred between clade 2.3.4.4b H5N8 and Eurasian low pathogenicity avian influenza viruses circulating in wild birds. Dissemination to South Korea during the 2017 fall migratory season followed.

Clade 2.3.4.4 H5 highly pathogenic avian influenza viruses (HPAIVs) have evolved by reassortment with different neuraminidase (NA) and internal genes of prevailing low pathogenicity avian influenza viruses (LPAIVs) and other HPAIVs to generate new genotypes and further evolved into genetic subgroups A–D since 2014 (*1*). Among these, subgroups A and B viruses were disseminated over vast geographic regions by migratory wild birds (*2*,*3*). Subgroup B influenza A(H5N8) viruses were detected in Qinghai Lake, China, and Uvs-Nuur Lake, Russia, during May–June 2016 (Qinghai/Uvs-like), followed by the identification of reassortant viruses in multiple Eurasian countries (*4*–*6*). Recently, subgroup B H5N6 viruses were isolated from birds in Greece during February 2017 and England, Germany, the Netherlands, Japan, and Taiwan during winter 2017–18 (*7*,*8*).

During December 2017–January 2018 in South Korea, we isolated 6 H5N6 HPAIVs from 231 fecal samples of wild birds collected from the banks of the Cheongmi-cheon River (37°06′56.9′′N, 127°25′18.3′′E) and 34 from 222 fecal samples collected from the banks of the Gokgyo-cheon River (36°45′12.3″N, 127°07′12.7″E) (Technical Appendix 1). These wild bird habitats are wintering sites of migratory waterfowl, including mallard (*Anas platyrhynchos*), spot-billed duck (*Anas poecilorhyncha*), Mandarin duck (*Aix galericulata*), and common teal (*Anas crecca*). The Gokgyo-cheon River is a major habitat site for Mandarin ducks, and numerous HPAIVs were detected in fecal samples from Mandarin ducks during 2011, 2015, and 2016 (*9*). We identified avian influenza virus–positive fecal samples from 38 Mandarin ducks and 2 mallards, based on DNA barcoding technique (*10*). We performed full-length genome sequencing and comparative phylogenetic analysis on 19 of the 40 isolates (Technical Appendix 1; Technical Appendix 2).

All H5N6 isolates shared high nucleotide sequence identities in all 8 gene segments (99.58%–100%) and were identified as HPAIVs based on the presence of multiple basic amino acids at the HA proteolytic cleavage site (PLREKRRKR/G). Searches of the GISAID (https://www.gisaid.org) and BLAST (https://blast.ncbi.nlm.nih.gov/Blast.cgi) databases indicated that all 8 genomes had the highest nucleotide identity with A/Great_Black-backed_Gull/Netherlands/1/2017 (Netherlands/1) clade 2.3.4.4 subgroup B H5N6 strain from December 2017 (99.17%–99.79%), rather than subgroup B H5N6 viruses from Japan and Taiwan collected during December 2017 (97.18%–99.27%).

In phylogenetic analysis, we identified 2 genotypes of subgroup B H5N6 viruses (Technical Appendix 1 Figures 1, 2): genotypes B.N6.1 and B.N6.2. The genotype B.N6.1 viruses were identified from South Korea, Japan, Taiwan, Greece, and the Netherlands (Netherlands/1 strain), and the genotype B.N6.2 viruses were detected from England, Germany, and the Netherlands. For genotype B.N6.1, all genes except NA clustered with H5N8 HPAIV of previously reported genotypes, H5N8-NL cluster I in the Netherlands (*6*), Ger-11-16 in Germany (*5*), and Duck/Poland/82a/16-like in Italy (*4*). The NA gene clustered with LPAIVs circulating in wild birds in Eurasia and separated into 2 clusters, suggesting the potential for >2 independent reassortment events between H5N8 virus and unidentified wild bird origin N6 segments. Consistent clustering of South Korea isolates with the Netherlands/1 strain in maximum-likelihood (ML) phylogenies for each gene supported by high ML bootstrap values (86–100) suggests their close relationship. The genotype B.N6.2 viruses had different polymerase basic 2 (PB2) and polymerase acidic (PA) genes from genotype B.N6.1. The polymerase basic 2 gene probably originated from other LPAIVs, and a polymerase acidic gene originated from H5N8-NL cluster II genotype (*6*,*7*). The phylogenetic network and ML analysis suggest that H5N6 viruses have evolved from subgroup B H5N8 viruses into 3 independent pathways, detected in Greece, Europe/South Korea, and Japan/Taiwan (Technical Appendix 1 Figures 2, 3).

The time of most recent common ancestry (tMRCA) for each gene of genotype B.N6.1 H5N6 viruses isolated during winter 2017–18 in Eurasia, except for the NA gene, ranged from January 2016 to October 2016, suggesting that genotype B.N6.1 viruses diverged during the previous year. The tMRCA of the NA gene was September 2015 (95% highest posterior density August 2014–August 2016). The tMRCA of the NA gene has wide 95% highest posterior density range because only a few recent N6 genes of LPAIVs were available in databases for analysis. The tMRCA for each gene of H5N6 HPAIVs identified in South Korea ranged from July through September 2017, suggesting that ancestors of these viruses emerged among wild birds during or after summer 2017, possibly at the breeding and molting sites in the Palearctic region (Table; Technical Appendix 1 Figure 4). Detection of H5N6 HPAIV from fecal samples of wild birds in South Korea during the 2017–18 wintering season and our phylogenetic analysis suggest that the viruses had moved through wild birds during the fall migration season.

**Table:** Time to most recent common ancestor for each gene segment of genotype B.N6.1 influenza A(H5N6) viruses isolated in South Korea, December 2017–January 2018*

Gene	South Korea isolates, node 1			South Korea and Europe isolates, node 2			South Korea, Europe, Japan, Taiwan, and Greece isolates, node 3	
Mean	95% HPD	Mean	95% HPD	Mean	95% HPD
PB2	2017 Sep	2017 Jul–Oct		2017 May	2016 Dec–2017 Sep		2016 Mar	2015 Oct–2016 Jun
PB1	2017 Sep	2017 Jul–Oct		2017 May	2017 Feb–Aug		2016 Jul	2016 May–Aug
PA	2017 Sep	2017 Aug–Oct		2017 Jul	2017 Apr–Sep		2016 Oct	2016 Jul–Dec
HA	2017 Sep	2017 Jul–Oct		2017 May	2017 Feb–Jul		2016 Mar	2015 Dec–2016 May
NP	2017 Jul	2017 Apr–Sep		2017 Mar	2016 Nov–2017 Jun		2016 Jan	2015 Jul–2016 May
NA	2017 Jul	2017 Mar–Sep		2017 Feb	2016 Jul–2017 Jul		2015 Sep	2014 Aug–2016 Aug
M	2017 Aug	2017 May–Oct		2017 May	2017 Jan–Aug		2016 Mar	2016 Jan–May
NS	2017 Jul	2017 Apr–Oct		2017 Mar	2016 Nov–2017 Jun		2016 Feb	2015 Oct–2016 Jun

*Nodes of the temporally structured maximum clade credibility phylogenetic tree (Technical Appendix Figure 4. HA, hemagglutinin; HPD, highest posterior density; M, matrix; NA, neuraminidase; NP, nucleoprotein; NS, nonstructural; PA, polymerase acidic; PB, polymerase basic.

On the basis of our data and migratory pattern of birds, we estimate that H5N6 viruses possibly descended from H5N8 viruses circulating during 2016–17, reaching breeding regions of wild birds during early 2017, followed by dissemination into Europe and East Asia during the fall migration. Enhanced surveillance in wild birds is needed for early detection of new introductions of HPAIV and to trace the transmission route of HPAIV.

Technical Appendix 1 Further details on study of new reassortant clade 2.3.4.4b avian influenza A(H5N6) virus in wild birds, South Korea, 2017–2018.

Technical Appendix 2 Influenza virus isolates used in the study of new reassortant clade 2.3.4.4b avian influenza A(H5N6) virus in wild birds, South Korea, 2017–2018.
