# Supplementary material for: New Reassortant Clade 2.3.4.4b Avian Influenza A(H5N6) Virus in Wild Birds, South Korea, 2017–18
Source: Emerg Infect Dis. 2018 Oct;24(10):1953–5. doi: 10.3201/eid2410.180461 (PMC6154165; doi:10.3201/eid2410.180461)
Supplement: Technical Appendix 1 — Further details on study of new reassortant clade 2.3.4.4b avian influenza A(H5N6) virus in wild birds, South Korea, 2017–2018. [file 18-0461-Techapp-s1.pdf]

Article DOI: https://doi.org/10.3201/eid2410.180461

New Reassortant Clade 2.3.4.4b Avian Influenza A(H5N6) Virus in Wild Birds, South Korea, 2017–2018

Technical Appendix

Methods

Virus Isolation

Fecal samples were tested for influenza A virus by egg inoculation using 9–1-day-old specific-pathogen-free embryonating chicken eggs. After a 72-h incubation period, allantoic fluids were harvested from an axenic medium, and undiluted allantoic fluids were tested for hemagglutinin (HA) activity. For the definitive diagnosis of influenza A virus, RNA was extracted from HA-positive allantoic fluid using an RNeasy Kit (QIAGEN, Valencia, CA, USA) according to the manufacturer's instructions. The extracted RNA was tested for the influenza A virus matrix gene by real-time reverse transcription PCR (RT-PCR) as described previously (*1*). The hosts of the positive fecal sample were identified as Mandarin ducks (*Aix galericulata*) and mallards (*Anas platyrhynchos*) using DNA barcoding techniques as previously described (*2*).

Full-genome Sequencing

Full-length genome sequencing was performed for 19 of 40 isolates (5 from Cheongmi-cheon River collected on 22 December 2017, 13 from Gokgyo-cheon River collected on 23 December 2017, and 1 from Gokgyo-cheon River collected on 18 January 2018). For molecular analysis, RNA was extracted using the RNeasy kit (QIAGEN) according to the manufacturer's instructions. The 8 genes of each virus were amplified using 1-step RT-PCR. The RT-PCR amplicons (2 μg) of all 8 gene segments was used to prepare Ion Fragment sequencing libraries (Life Technologies, Carlsbad, CA, USA) according to the manufacturer's instructions. Briefly, amplicons were loaded onto beads, and emulsion PCR was conducted before sequencing with an Ion 318 chip on an Ion Torrent Personal Genome Machine. De novo and directed assembly of genome sequences were performed using Geneious R9 software (http://www.geneious.com). We

provided the genome sequences in the EpiFlu database of Global Initiative on Sharing All Influenza Data (GISAID; https://www.gisaid.org), under accession nos. EPI1190336–40, EPI1190342–61, EPI1190381, EPI1190386, EPI1190392, EPI1190410, EPI1190418, EPI1190426, EPI1190426, EPI1190433, EPI1190446, EPI1190498, EPI1190503, EPI1190508, EPI1190514–19, EPI1200474–509, EPI1200511–87.

Phylogenetic Analysis

For phylogenetic analysis, complete coding regions were aligned using MAFFT (https://mafft.cbrc.jp/alignment/software/), and manual editing and tree reconstruction were conducted using Geneious 8 software (https://www.geneious.com/). The sequence of clade 2.3.4.4 H5 highly pathogenic avian influenza viruses representing each subgroup and low pathogenic avian influenza virus strains related to new reassortment of H5N6 highly pathogenic avian influenza viruses in 2017–18 were selected from GISAID Epiflu and GenBank (https://www.ncbi.nlm.nih.gov/genomes/FLU) database as a reference for phylogenetic tree analysis. A maximum-likelihood tree was estimated by the MEGA7 software using the Hasegawa-Kishino-Yano model (*3*) of nucleotide substitution with gamma-distributed rate variation among sites with 4 rate categories. Statistical analysis of the phylogenetic tree was performed by bootstrap analysis on 1,000 replicates. The percentages of replicate trees (>70%) in which the associated taxa clustered together in the bootstrap test are shown next to the branches.

To better visualize the genetic relatedness of viruses, HA gene was analyzed using the median joining method implemented by NETWORK ver. 5.0 with epsilon set to 0. Nucleotide sequences of complete protein coding sequence region of subgroup B H5N6 and H5N8 identified from Asia and Europe in 2016 and 2017 (n = 250) were retrieved from GISAID Epiflu database on February 13, 2018. The sequences were pruned by using the online software cd-hit (*4*) at 97% homology to filter-out 66 representative sequences. The nucleotide sequences of HA segment obtained in this study (n = 19) were added to the dataset. A total of 85 nt sequences were aligned using MAFFT, and alignments were manually edited in Geneious 8 software (https://www.geneious.com/).

For estimation of the time to the most recent common ancestor, Bayesian analysis was performed for all 8 gene segments using BEAST version 1.8.4 (http://beast.bio.ed.ac.uk). We used an Hasegawa-Kishino-Yano substitution model with 4 gamma categories and specified an

uncorrelated lognormal relaxed clock and GMRF Bayesian skyride tree prior for each segment. A Markov chain Monte Carlo method was used with 50 million chain lengths to draw inference under this model. A maximum clade credibility tree with common ancestor height was generated for each dataset using TreeAnnotator version 1.8.4 after 10% burn-in. The maximum clade credibility trees were visualized using the program FigTree v1.4.2.

References

1. Spackman E, Senne DA, Bulaga LL, Myers TJ, Perdue ML, Garber LP, et al. Development of real-time RT-PCR for the detection of avian influenza virus. Avian Dis. 2003;47(Suppl):1079–82. PubMed http://dx.doi.org/10.1637/0005-2086-47.s3.1079

2. Lee DH, Lee HJ, Lee YJ, Kang HM, Jeong OM, Kim MC, et al. DNA barcoding techniques for avian influenza virus surveillance in migratory bird habitats. J Wildl Dis. 2010;46:649–54. PubMed http://dx.doi.org/10.7589/0090-3558-46.2.649

3. Hasegawa M, Kishino H, Yano T. Dating of the human-ape splitting by a molecular clock of mitochondrial DNA. J Mol Evol. 1985;22:160–74. PubMed http://dx.doi.org/10.1007/BF02101694

4. Huang Y, Niu B, Gao Y, Fu L, Li W. CD-HIT Suite: a web server for clustering and comparing biological sequences. Bioinformatics. 2010;26:680–2. PubMed http://dx.doi.org/10.1093/bioinformatics/btq003

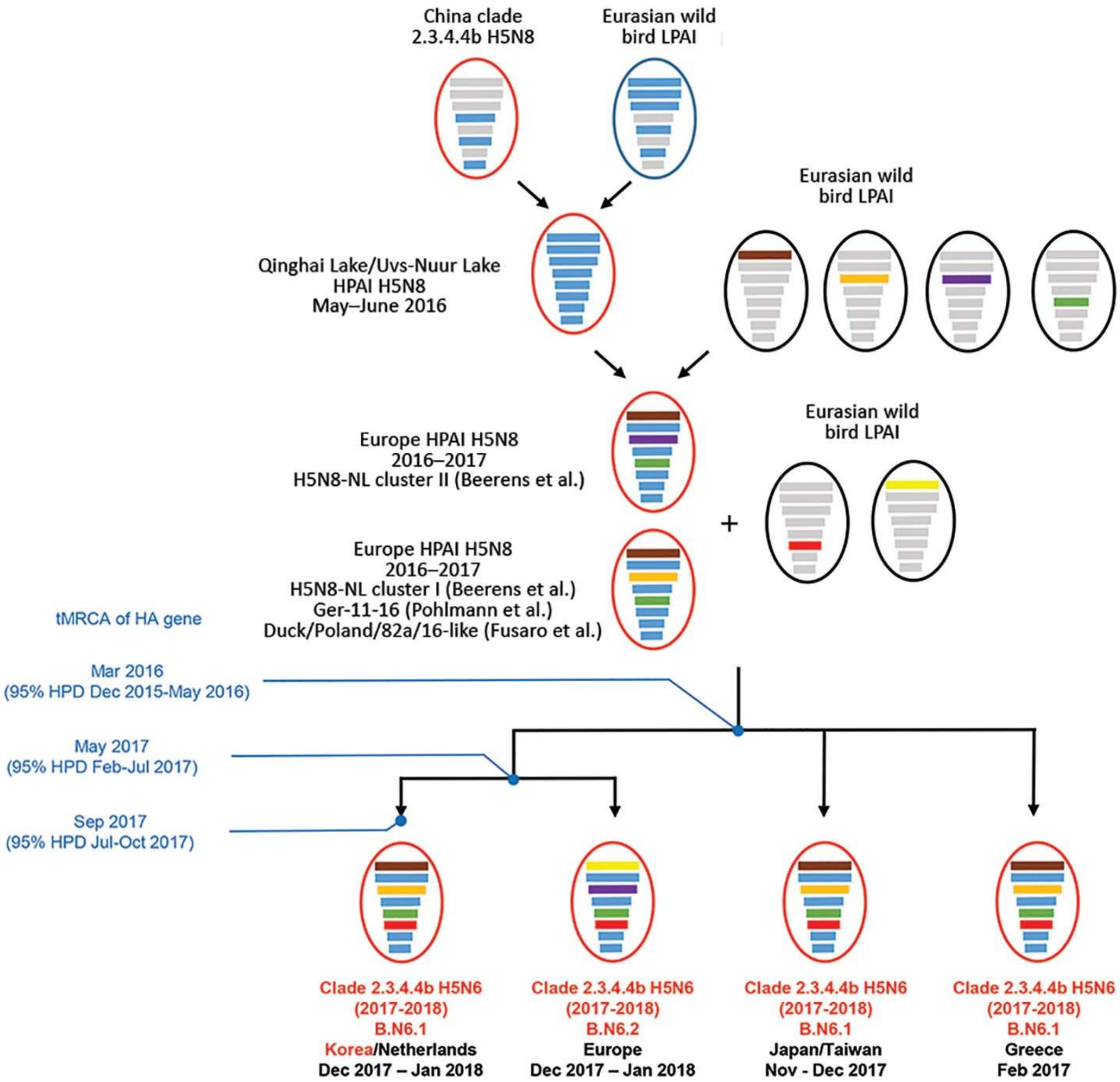

Technical Appendix 1 Figure 1. Proposed reassortment events leading to the new subgroup B influenza A(H5N6) viruses. Eight gene segments in each of the schematic virus particles are arranged from top to bottom to represent the PB2, PB1, PA, HA, NP, NA, M, and NS genes. The circled HPAI viruses are colored in red, and unknown LPAI viruses are colored in black. For each gene segment, different phylogenetic groupings are in different colors. The mean time to the most recent common ancestor of HA gene are shown at the node. HA, hemagglutinin; HPAI, highly pathogenic avian influenza; HPD, highest posterior density; LPAI, low pathogen avian influenza; M, matrix; NA, neuraminidase; NP, nucleoprotein; NS, nonstructural; PA, polymerase acidic; PB, polymerase basic; tMCRA, time to the most recent common ancestor.

A

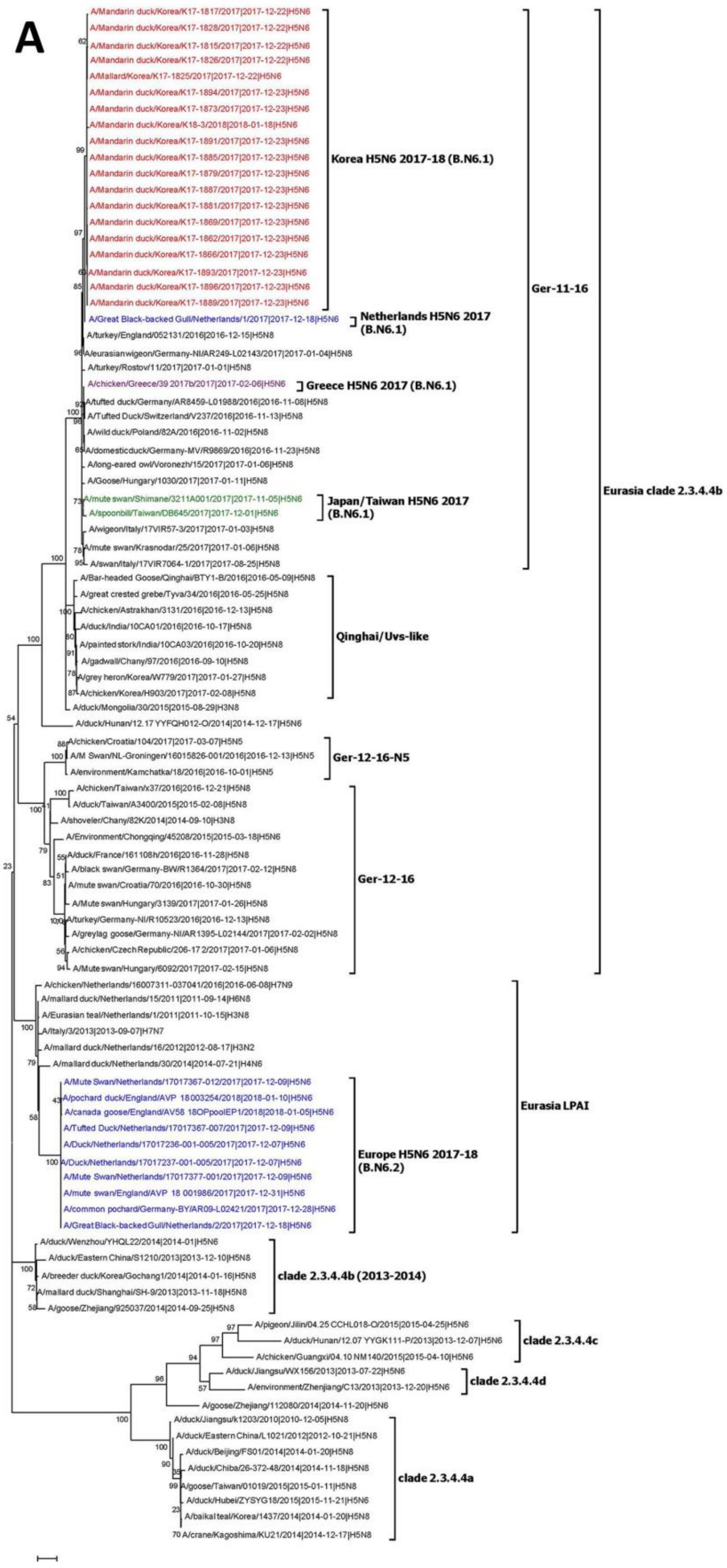

A/Mandarin duck/Korea/K17-1817/2017|2017-12-22|H5N6
A/Mandarin duck/Korea/K17-1828/2017|2017-12-22|H5N6
A/Mandarin duck/Korea/K17-1815/2017|2017-12-22|H5N6
A/Mandarin duck/Korea/K17-1826/2017|2017-12-22|H5N6
A/Mallard/Korea/K17-1825/2017|2017-12-22|H5N6
A/Mandarin duck/Korea/K17-1894/2017|2017-12-23|H5N6
A/Mandarin duck/Korea/K17-1873/2017|2017-12-23|H5N6
A/Mandarin duck/Korea/K18-3/2018|2018-01-18|H5N6
A/Mandarin duck/Korea/K17-1891/2017|2017-12-23|H5N6
A/Mandarin duck/Korea/K17-1885/2017|2017-12-23|H5N6
A/Mandarin duck/Korea/K17-1879/2017|2017-12-23|H5N6
A/Mandarin duck/Korea/K17-1887/2017|2017-12-23|H5N6
A/Mandarin duck/Korea/K17-1881/2017|2017-12-23|H5N6
A/Mandarin duck/Korea/K17-1869/2017|2017-12-23|H5N6
A/Mandarin duck/Korea/K17-1862/2017|2017-12-23|H5N6
A/Mandarin duck/Korea/K17-1866/2017|2017-12-23|H5N6
A/Mandarin duck/Korea/K17-1893/2017|2017-12-23|H5N6
A/Mandarin duck/Korea/K17-1896/2017|2017-12-23|H5N6
A/Mandarin duck/Korea/K17-1889/2017|2017-12-23|H5N6
Korea H5N6 2017-18 (B.N6.1)
A/Great Black-backed Gull/Netherlands/1/2017|2017-12-18|H5N6
Netherlands H5N6 2017 (B.N6.1)
A/turkey/England/052131/2016|2016-12-15|H5N8
A/eurasianwigeon/Germany-NI/AR249-L02143/2017|2017-01-04|H5N8
A/turkey/Rostov/11/2017|2017-01-01|H5N8
A/chicken/Greece/39 2017b/2017|2017-02-06|H5N6
Greece H5N6 2017 (B.N6.1)
A/tufted duck/Germany/AR8459-L01988/2016|2016-11-08|H5N8
A/Tufted Duck/Switzerland/V237/2016|2016-11-13|H5N8
A/wild duck/Poland/82A/2016|2016-11-02|H5N8
A/domesticduck/Germany-MV/R9869/2016|2016-11-23|H5N8
A/long-eared owl/Voronezh/15/2017|2017-01-06|H5N8
A/Goose/Hungary/1030/2017|2017-01-11|H5N8
A/mute swan/Shimane/3211A001/2017|2017-11-05|H5N6
A/spoonbill/Taiwan/DB645/2017|2017-12-01|H5N6
Japan/Taiwan H5N6 2017 (B.N6.1)
A/wigeon/Italy/17VIR57-3/2017|2017-01-03|H5N8
A/mute swan/Krasnodar/25/2017|2017-01-06|H5N8
A/swan/Italy/17VIR7064-1/2017|2017-08-25|H5N8
Ger-11-16
A/Bar-headed Goose/Qinghai/BTY1-B/2016|2016-05-09|H5N8
A/great crested grebe/Tyva/34/2016|2016-05-25|H5N8
A/chicken/Astrakhan/3131/2016|2016-12-13|H5N8
A/duck/India/10CA01/2016|2016-10-17|H5N8
A/painted stork/India/10CA03/2016|2016-10-20|H5N8
A/gadwall/Chany/97/2016|2016-09-10|H5N8
A/grey heron/Korea/W779/2017|2017-01-27|H5N8
A/chicken/Korea/H903/2017|2017-02-08|H5N8
Qinghai/Uvs-like
A/duck/Mongolia/30/2015|2015-08-29|H3N8
A/duck/Hunan/12.17 YYFQH012-O/2014|2014-12-17|H5N6
A/chicken/Croatia/104/2017|2017-03-07|H5N5
A/M Swan/NL-Groningen/16015826-001/2016|2016-12-13|H5N5
A/environment/Kamchatka/18/2016|2016-10-01|H5N5
Ger-12-16-N5
A/chicken/Taiwan/x37/2016|2016-12-21|H5N8
A/duck/Taiwan/A3400/2015|2015-02-08|H5N8
A/shoveler/Chany/82K/2014|2014-09-10|H3N8
A/Environment/Chongqing/45208/2015|2015-03-18|H5N6
A/duck/France/161108h/2016|2016-11-28|H5N8
A/black swan/Germany-BW/R1364/2017|2017-02-12|H5N8
A/mute swan/Croatia/70/2016|2016-10-30|H5N8
A/Mute swan/Hungary/3139/2017|2017-01-26|H5N8
A/turkey/Germany-NI/R10523/2016|2016-12-13|H5N8
A/greylag goose/Germany-NI/AR1395-L02144/2017|2017-02-02|H5N8
A/chicken/Czech Republic/206-17 2/2017|2017-01-06|H5N8
A/Mute swan/Hungary/6092/2017|2017-02-15|H5N8
Ger-12-16
Eurasia clade 2.3.4.4b
A/chicken/Netherlands/16007311-037041/2016|2016-06-08|H7N9
A/mallard duck/Netherlands/15/2011|2011-09-14|H6N8
A/Eurasian teal/Netherlands/1/2011|2011-10-15|H3N8
A/Italy/3/2013|2013-09-07|H7N7
A/mallard duck/Netherlands/16/2012|2012-08-17|H3N2
A/mallard duck/Netherlands/30/2014|2014-07-21|H4N6
A/Mute Swan/Netherlands/17017367-012/2017|2017-12-09|H5N6
A/pochard duck/England/AVP 18003254/2018|2018-01-10|H5N6
A/canada goose/England/AV58 18OPpoolEP1/2018|2018-01-05|H5N6
A/Tufted Duck/Netherlands/17017367-007/2017|2017-12-09|H5N6
A/Duck/Netherlands/17017236-001-005/2017|2017-12-07|H5N6
A/Duck/Netherlands/17017237-001-005/2017|2017-12-07|H5N6
A/Mute Swan/Netherlands/17017377-001/2017|2017-12-09|H5N6
A/mute swan/England/AVP 18 001986/2017|2017-12-31|H5N6
A/common pochard/Germany-BY/AR09-L02421/2017|2017-12-28|H5N6
A/Great Black-backed Gull/Netherlands/2/2017|2017-12-18|H5N6
Europe H5N6 2017-18 (B.N6.2)
Eurasia LPAI
A/duck/Wenzhou/YHQL22/2014|2014-01|H5N6
A/duck/Eastern China/S1210/2013|2013-12-10|H5N8
A/breeder duck/Korea/Gochang1/2014|2014-01-16|H5N8
A/mallard duck/Shanghai/SH-9/2013|2013-11-18|H5N8
A/goose/Zhejiang/925037/2014|2014-09-25|H5N8
clade 2.3.4.4b (2013-2014)
A/pigeon/Jilin/04.25 CCHL018-O/2015|2015-04-25|H5N6
A/duck/Hunan/12.07 YYGK111-P/2013|2013-12-07|H5N6
A/chicken/Guangxi/04.10 NM140/2015|2015-04-10|H5N6
clade 2.3.4.4c
A/duck/Jiangsu/WX156/2013|2013-07-22|H5N6
A/environment/Zhenjiang/C13/2013|2013-12-20|H5N6
clade 2.3.4.4d
A/goose/Zhejiang/112080/2014|2014-11-20|H5N6
A/duck/Jiangsu/k1203/2010|2010-12-05|H5N8
A/duck/Eastern China/L1021/2012|2012-10-21|H5N8
A/duck/Beijing/FS01/2014|2014-01-20|H5N8
A/duck/Chiba/26-372-48/2014|2014-11-18|H5N8
A/goose/Taiwan/01019/2015|2015-01-11|H5N8
A/duck/Hubei/ZYSYG18/2015|2015-11-21|H5N6
A/baikal teal/Korea/1437/2014|2014-01-20|H5N8
A/crane/Kagoshima/KU21/2014|2014-12-17|H5N8
clade 2.3.4.4a

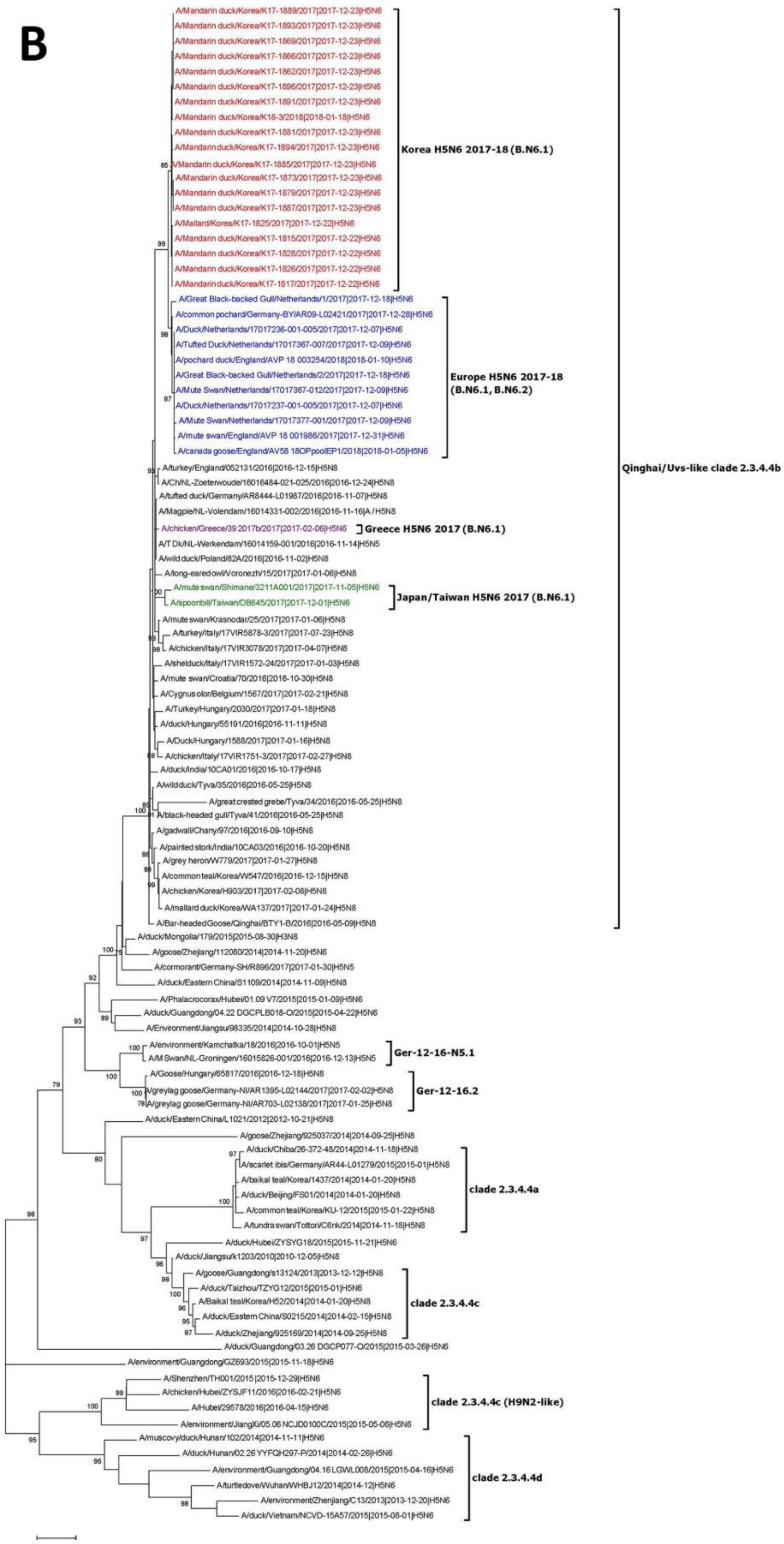
B
A/Mandarin duck/Korea/K17-1889/2017|2017-12-23|H5N6
A/Mandarin duck/Korea/K17-1893/2017|2017-12-23|H5N6
A/Mandarin duck/Korea/K17-1869/2017|2017-12-23|H5N6
A/Mandarin duck/Korea/K17-1866/2017|2017-12-23|H5N6
A/Mandarin duck/Korea/K17-1862/2017|2017-12-23|H5N6
A/Mandarin duck/Korea/K17-1896/2017|2017-12-23|H5N6
A/Mandarin duck/Korea/K17-1891/2017|2017-12-23|H5N6
A/Mandarin duck/Korea/K18-3/2018|2018-01-18|H5N6
A/Mandarin duck/Korea/K17-1881/2017|2017-12-23|H5N6
A/Mandarin duck/Korea/K17-1894/2017|2017-12-23|H5N6
A/Mandarin duck/Korea/K17-1885/2017|2017-12-23|H5N6
A/Mandarin duck/Korea/K17-1873/2017|2017-12-23|H5N6
A/Mandarin duck/Korea/K17-1879/2017|2017-12-23|H5N6
A/Mandarin duck/Korea/K17-1887/2017|2017-12-23|H5N6
A/Mallard/Korea/K17-1825/2017|2017-12-22|H5N6
A/Mandarin duck/Korea/K17-1815/2017|2017-12-22|H5N6
A/Mandarin duck/Korea/K17-1828/2017|2017-12-22|H5N6
A/Mandarin duck/Korea/K17-1826/2017|2017-12-22|H5N6
A/Mandarin duck/Korea/K17-1817/2017|2017-12-22|H5N6
Korea H5N6 2017-18 (B.N6.1)
A/Great Black-backed Gull/Netherlands/1/2017|2017-12-18|H5N6
A/common pochard/Germany-BY/AR09-L02421/2017|2017-12-28|H5N6
A/Duck/Netherlands/17017236-001-005/2017|2017-12-07|H5N6
A/Tufted Duck/Netherlands/17017367-007/2017|2017-12-09|H5N6
A/pochard duck/England/AVP 18 003254/2018|2018-01-10|H5N6
A/Great Black-backed Gull/Netherlands/2/2017|2017-12-18|H5N6
A/Mute Swan/Netherlands/17017367-012/2017|2017-12-09|H5N6
A/Duck/Netherlands/17017237-001-005/2017|2017-12-07|H5N6
A/Mute Swan/Netherlands/17017377-001/2017|2017-12-09|H5N6
A/mute swan/England/AVP 18 001986/2017|2017-12-31|H5N6
A/canada goose/England/AV58 18OPpoolEP1/2018|2018-01-05|H5N6
Europe H5N6 2017-18 (B.N6.1, B.N6.2)
A/turkey/England/052131/2016|2016-12-15|H5N8
A/Ch/NL-Zoeterwoude/16016484-021-025/2016|2016-12-24|H5N8
A/tufted duck/Germany/AR8444-L01987/2016|2016-11-07|H5N8
A/Magpie/NL-Volendam/16014331-002/2016|2016-11-16|A /H5N8
A/chicken/Greece/39 2017b/2017|2017-02-06|H5N6
Greece H5N6 2017 (B.N6.1)
A/T Dk/NL-Werkendam/16014159-001/2016|2016-11-14|H5N5
A/wild duck/Poland/82A/2016|2016-11-02|H5N8
A/long-eared owl/Voronezh/15/2017|2017-01-06|H5N8
A/mute swan/Shimane/3211A001/2017|2017-11-05|H5N6
A/spoonbill/Taiwan/DB645/2017|2017-12-01|H5N6
Japan/Taiwan H5N6 2017 (B.N6.1)
A/mute swan/Krasnodar/25/2017|2017-01-06|H5N8
A/turkey/Italy/17VIR5878-3/2017|2017-07-23|H5N8
A/chicken/Italy/17VIR3078/2017|2017-04-07|H5N8
A/shelduck/Italy/17VIR1572-24/2017|2017-01-03|H5N8
A/mute swan/Croatia/70/2016|2016-10-30|H5N8
A/Cygnus olor/Belgium/1567/2017|2017-02-21|H5N8
A/Turkey/Hungary/2030/2017|2017-01-18|H5N8
A/duck/Hungary/55191/2016|2016-11-11|H5N8
A/Duck/Hungary/1588/2017|2017-01-16|H5N8
A/chicken/Italy/17VIR1751-3/2017|2017-02-27|H5N8
A/duck/India/10CA01/2016|2016-10-17|H5N8
A/wild duck/Tyva/35/2016|2016-05-25|H5N8
A/great crested grebe/Tyva/34/2016|2016-05-25|H5N8
A/black-headed gull/Tyva/41/2016|2016-05-25|H5N8
A/gadwall/Chany/97/2016|2016-09-10|H5N8
A/painted stork/India/10CA03/2016|2016-10-20|H5N8
A/grey heron/W779/2017|2017-01-27|H5N8
A/common teal/Korea/W547/2016|2016-12-15|H5N8
A/chicken/Korea/H903/2017|2017-02-08|H5N8
A/mallard duck/Korea/WA137/2017|2017-01-24|H5N8
A/Bar-headed Goose/Qinghai/BTY1-B/2016|2016-05-09|H5N8
Qinghai/Uvs-like clade 2.3.4.4b
A/duck/Mongolia/179/2015|2015-08-30|H3N8
A/goose/Zhejiang/112080/2014|2014-11-20|H5N6
A/cormorant/Germany-SH/R896/2017|2017-01-30|H5N5
A/duck/Eastern China/S1109/2014|2014-11-09|H5N8
A/Phalacrocorax/Hubei/01.09 V7/2015|2015-01-09|H5N6
A/duck/Guangdong/04.22 DGCPLB018-O/2015|2015-04-22|H5N6
A/Environment/Jiangsu/98335/2014|2014-10-28|H5N8
A/environment/Kamchatka/18/2016|2016-10-01|H5N5
A/M Swan/NL-Groningen/16015826-001/2016|2016-12-13|H5N5
Ger-12-16-N5.1
A/Goose/Hungary/65817/2016|2016-12-18|H5N8
A/greylag goose/Germany-NI/AR1395-L02144/2017|2017-02-02|H5N8
A/greylag goose/Germany-NI/AR703-L02138/2017|2017-01-25|H5N8
Ger-12-16.2
A/duck/Eastern China/L1021/2012|2012-10-21|H5N8
A/goose/Zhejiang/925037/2014|2014-09-25|H5N8
A/duck/Chiba/26-372-48/2014|2014-11-18|H5N8
A/scarlet ibis/Germany/AR44-L01279/2015|2015-01|H5N8
A/baikal teal/Korea/1437/2014|2014-01-20|H5N8
A/duck/Beijing/FS01/2014|2014-01-20|H5N8
A/common teal/Korea/KU-12/2015|2015-01-22|H5N8
A/tundra swan/Tottori/C6nk/2014|2014-11-18|H5N8
clade 2.3.4.4a
A/duck/Hubei/ZYSYG18/2015|2015-11-21|H5N6
A/duck/Jiangsu/k1203/2010|2010-12-05|H5N8
A/goose/Guangdong/s13124/2013|2013-12-12|H5N8
A/duck/Taizhou/TZYG12/2015|2015-01|H5N6
A/Baikal teal/Korea/H52/2014|2014-01-20|H5N8
A/duck/Eastern China/S0215/2014|2014-02-15|H5N8
A/duck/Zhejiang/925169/2014|2014-09-25|H5N8
clade 2.3.4.4c
A/duck/Guangdong/03.26 DGCP077-O/2015|2015-03-26|H5N6
A/environment/Guangdong/GZ693/2015|2015-11-18|H5N6
A/Shenzhen/TH001/2015 |2015-12-29|H5N6
A/chicken/Hubei/ZYSJF11/2016|2016-02-21|H5N6
A/Hubei/29578/2016|2016-04-15|H5N6
A/environment/JiangXi/05.06 NCJD0100C/2015|2015-05-06|H5N6
clade 2.3.4.4c (H9N2-like)
A/muscovy/duck/Hunan/102/2014|2014-11-11|H5N6
A/duck/Hunan/02.26 YYFQH297-P/2014|2014-02-26|H5N6
A/environment/Guangdong/04.16 LGWL008/2015|2015-04-16|H5N6
A/turtledove/Wuhan/WHBJ12/2014|2014-12|H5N6
A/environment/Zhenjiang/C13/2013|2013-12-20|H5N6
A/duck/Vietnam/NCVD-15A57/2015|2015-08-01|H5N6
clade 2.3.4.4d

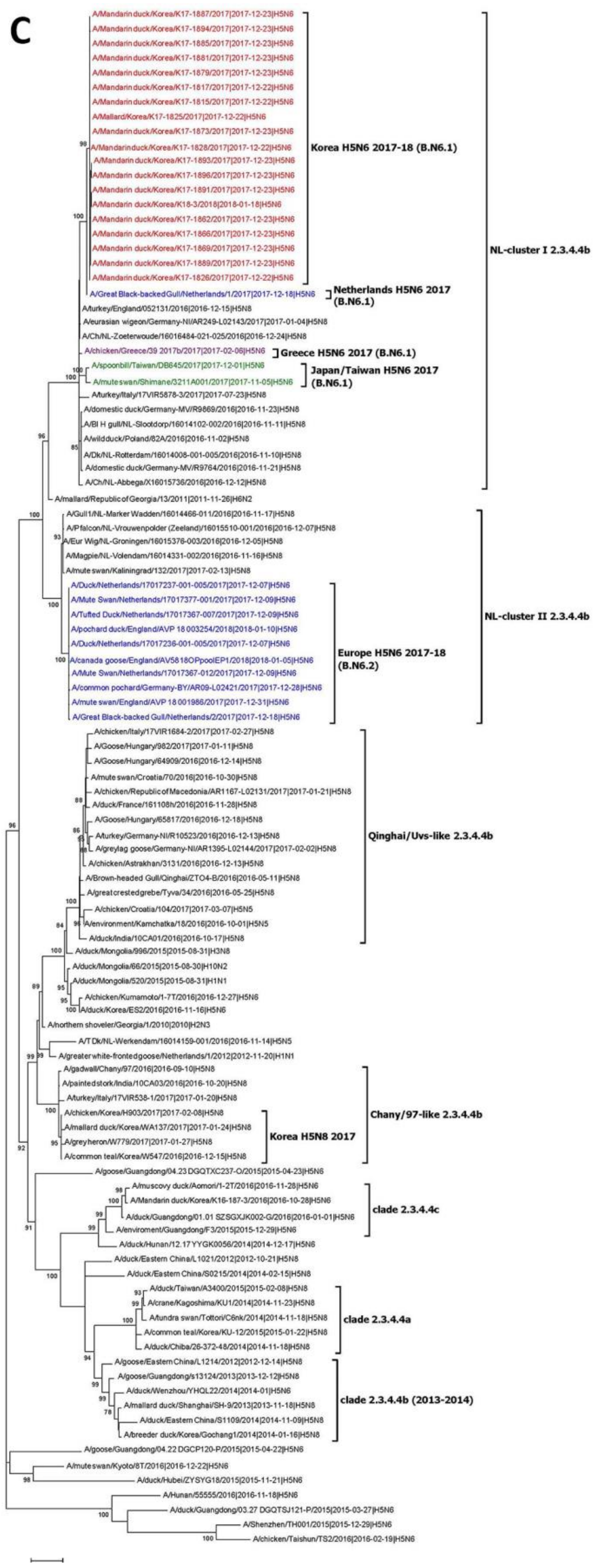
C
Korea H5N6 2017-18 (B.N6.1)
Netherlands H5N6 2017 (B.N6.1)
Greece H5N6 2017 (B.N6.1)
Japan/Taiwan H5N6 2017 (B.N6.1)
NL-cluster I 2.3.4.4b
Europe H5N6 2017-18 (B.N6.2)
NL-cluster II 2.3.4.4b
Qinghai/Uvs-like 2.3.4.4b
Chany/97-like 2.3.4.4b
Korea H5N8 2017
clade 2.3.4.4c
clade 2.3.4.4a
clade 2.3.4.4b (2013-2014)

D

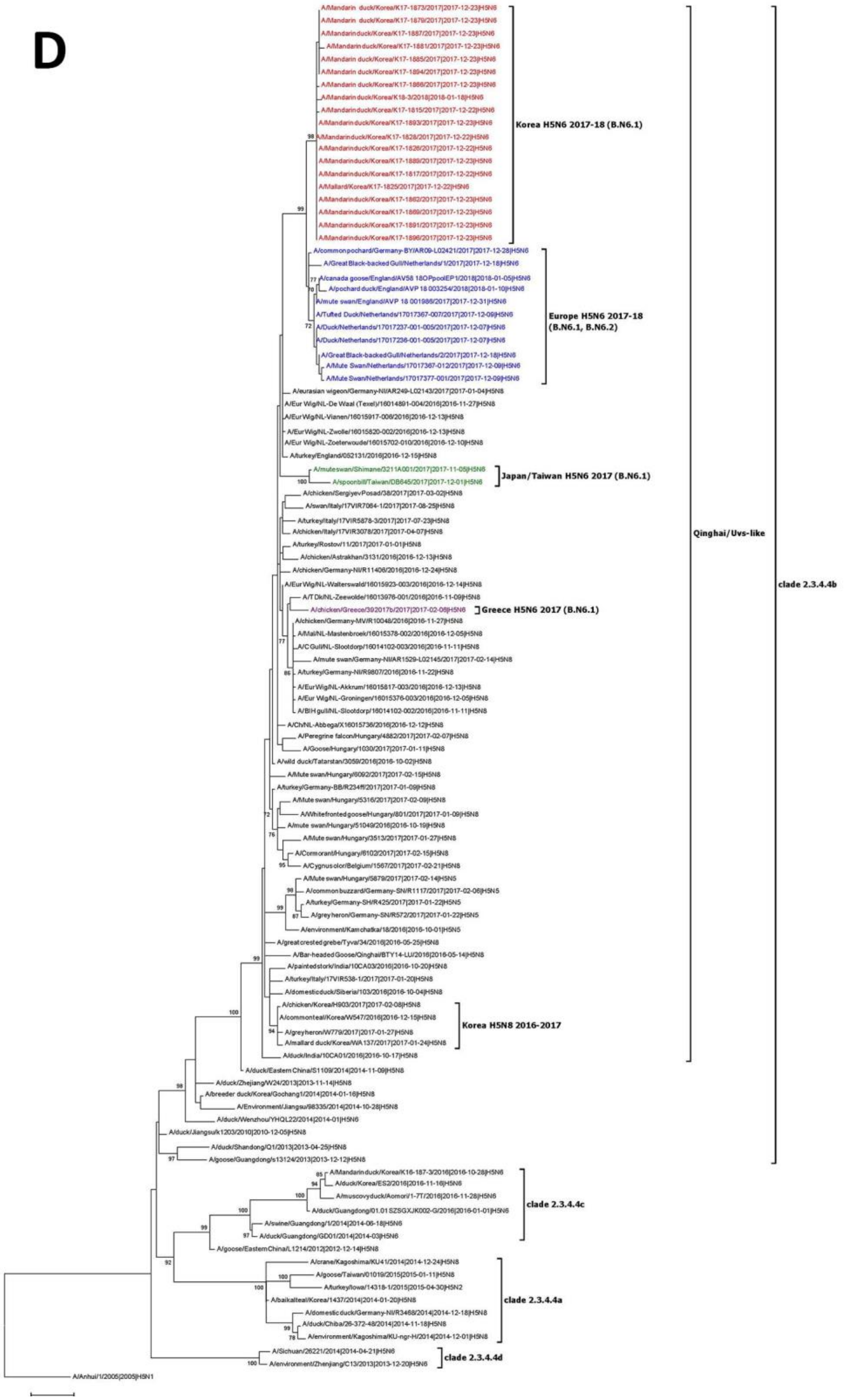
Korea H5N6 2017-18 (B.N6.1)
Europe H5N6 2017-18
(B.N6.1, B.N6.2)
Japan/Taiwan H5N6 2017 (B.N6.1)
Qinghai/Uvs-like
clade 2.3.4.4b
Greece H5N6 2017 (B.N6.1)
Korea H5N8 2016-2017
clade 2.3.4.4c
clade 2.3.4.4a
clade 2.3.4.4d

E

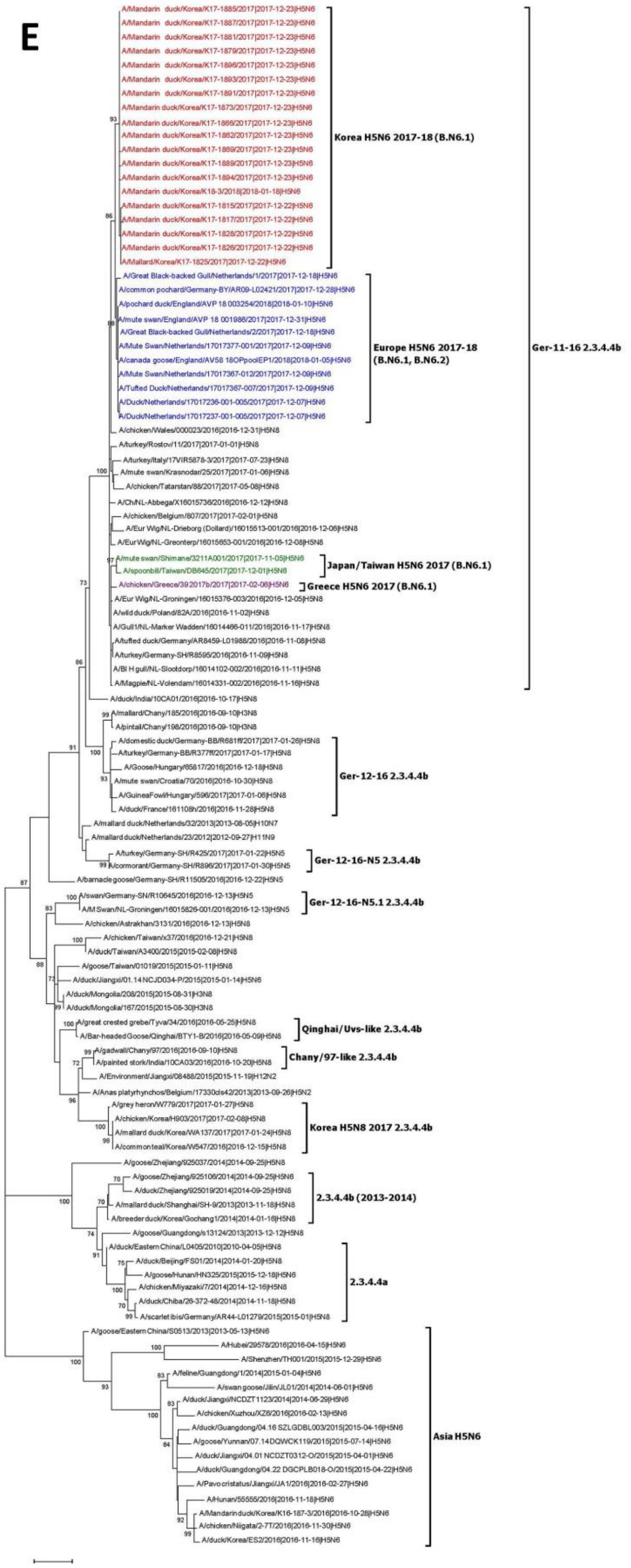
Korea H5N6 2017-18 (B.N6.1)
Europe H5N6 2017-18 (B.N6.1, B.N6.2)
Ger-11-16 2.3.4.4b
Japan/Taiwan H5N6 2017 (B.N6.1)
Greece H5N6 2017 (B.N6.1)
Ger-12-16 2.3.4.4b
Ger-12-16-N5 2.3.4.4b
Ger-12-16-N5.1 2.3.4.4b
Qinghai/Uvs-like 2.3.4.4b
Chany/97-like 2.3.4.4b
Korea H5N8 2017 2.3.4.4b
2.3.4.4b (2013-2014)
2.3.4.4a
Asia H5N6

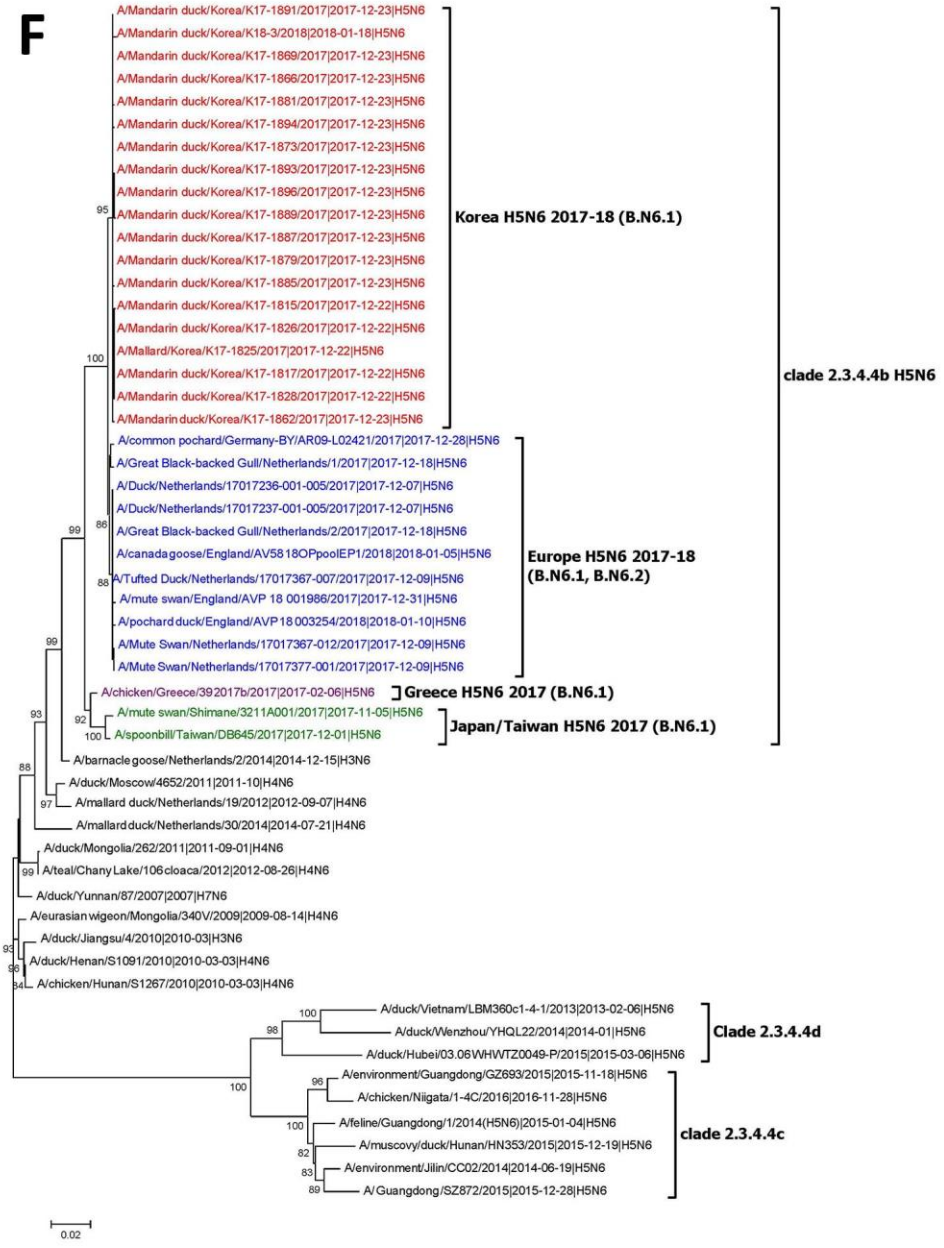
F
A/Mandarin duck/Korea/K17-1891/2017|2017-12-23|H5N6
A/Mandarin duck/Korea/K18-3/2018|2018-01-18|H5N6
A/Mandarin duck/Korea/K17-1869/2017|2017-12-23|H5N6
A/Mandarin duck/Korea/K17-1866/2017|2017-12-23|H5N6
A/Mandarin duck/Korea/K17-1881/2017|2017-12-23|H5N6
A/Mandarin duck/Korea/K17-1894/2017|2017-12-23|H5N6
A/Mandarin duck/Korea/K17-1873/2017|2017-12-23|H5N6
A/Mandarin duck/Korea/K17-1893/2017|2017-12-23|H5N6
A/Mandarin duck/Korea/K17-1896/2017|2017-12-23|H5N6
A/Mandarin duck/Korea/K17-1889/2017|2017-12-23|H5N6
A/Mandarin duck/Korea/K17-1887/2017|2017-12-23|H5N6
A/Mandarin duck/Korea/K17-1879/2017|2017-12-23|H5N6
A/Mandarin duck/Korea/K17-1885/2017|2017-12-23|H5N6
A/Mandarin duck/Korea/K17-1815/2017|2017-12-22|H5N6
A/Mandarin duck/Korea/K17-1826/2017|2017-12-22|H5N6
A/Mallard/Korea/K17-1825/2017|2017-12-22|H5N6
A/Mandarin duck/Korea/K17-1817/2017|2017-12-22|H5N6
A/Mandarin duck/Korea/K17-1828/2017|2017-12-22|H5N6
A/Mandarin duck/Korea/K17-1862/2017|2017-12-23|H5N6
Korea H5N6 2017-18 (B.N6.1)
A/common pochard/Germany-BY/AR09-L02421/2017|2017-12-28|H5N6
A/Great Black-backed Gull/Netherlands/1/2017|2017-12-18|H5N6
A/Duck/Netherlands/17017236-001-005/2017|2017-12-07|H5N6
A/Duck/Netherlands/17017237-001-005/2017|2017-12-07|H5N6
A/Great Black-backed Gull/Netherlands/2/2017|2017-12-18|H5N6
A/canada goose/England/AV5818OPpoolEP1/2018|2018-01-05|H5N6
A/Tufted Duck/Netherlands/17017367-007/2017|2017-12-09|H5N6
A/mute swan/England/AVP 18 001986/2017|2017-12-31|H5N6
A/pochard duck/England/AVP 18 003254/2018|2018-01-10|H5N6
A/Mute Swan/Netherlands/17017367-012/2017|2017-12-09|H5N6
A/Mute Swan/Netherlands/17017377-001/2017|2017-12-09|H5N6
Europe H5N6 2017-18
(B.N6.1, B.N6.2)
A/chicken/Greece/39 2017b/2017|2017-02-06|H5N6
Greece H5N6 2017 (B.N6.1)
A/mute swan/Shimane/3211A001/2017|2017-11-05|H5N6
A/spoonbill/Taiwan/DB645/2017|2017-12-01|H5N6
Japan/Taiwan H5N6 2017 (B.N6.1)
clade 2.3.4.4b H5N6
A/barnacle goose/Netherlands/2/2014|2014-12-15|H3N6
A/duck/Moscow/4652/2011|2011-10|H4N6
A/mallard duck/Netherlands/19/2012|2012-09-07|H4N6
A/mallard duck/Netherlands/30/2014|2014-07-21|H4N6
A/duck/Mongolia/262/2011|2011-09-01|H4N6
A/teal/Chany Lake/106 cloaca/2012|2012-08-26|H4N6
A/duck/Yunnan/87/2007|2007|H7N6
A/eurasian wigeon/Mongolia/340V/2009|2009-08-14|H4N6
A/duck/Jiangsu/4/2010|2010-03|H3N6
A/duck/Henan/S1091/2010|2010-03-03|H4N6
A/chicken/Hunan/S1267/2010|2010-03-03|H4N6
A/duck/Vietnam/LBM360c1-4-1/2013|2013-02-06|H5N6
A/duck/Wenzhou/YHQL22/2014|2014-01|H5N6
A/duck/Hubei/03.06WHWTZ0049-P/2015|2015-03-06|H5N6
Clade 2.3.4.4d
A/environment/Guangdong/GZ693/2015|2015-11-18|H5N6
A/chicken/Niigata/1-4C/2016|2016-11-28|H5N6
A/feline/Guangdong/1/2014(H5N6)|2015-01-04|H5N6
A/muscovy/duck/Hunan/HN353/2015|2015-12-19|H5N6
A/environment/Jilin/CC02/2014|2014-06-19|H5N6
A/Guangdong/SZ872/2015|2015-12-28|H5N6
clade 2.3.4.4c
0.02

G

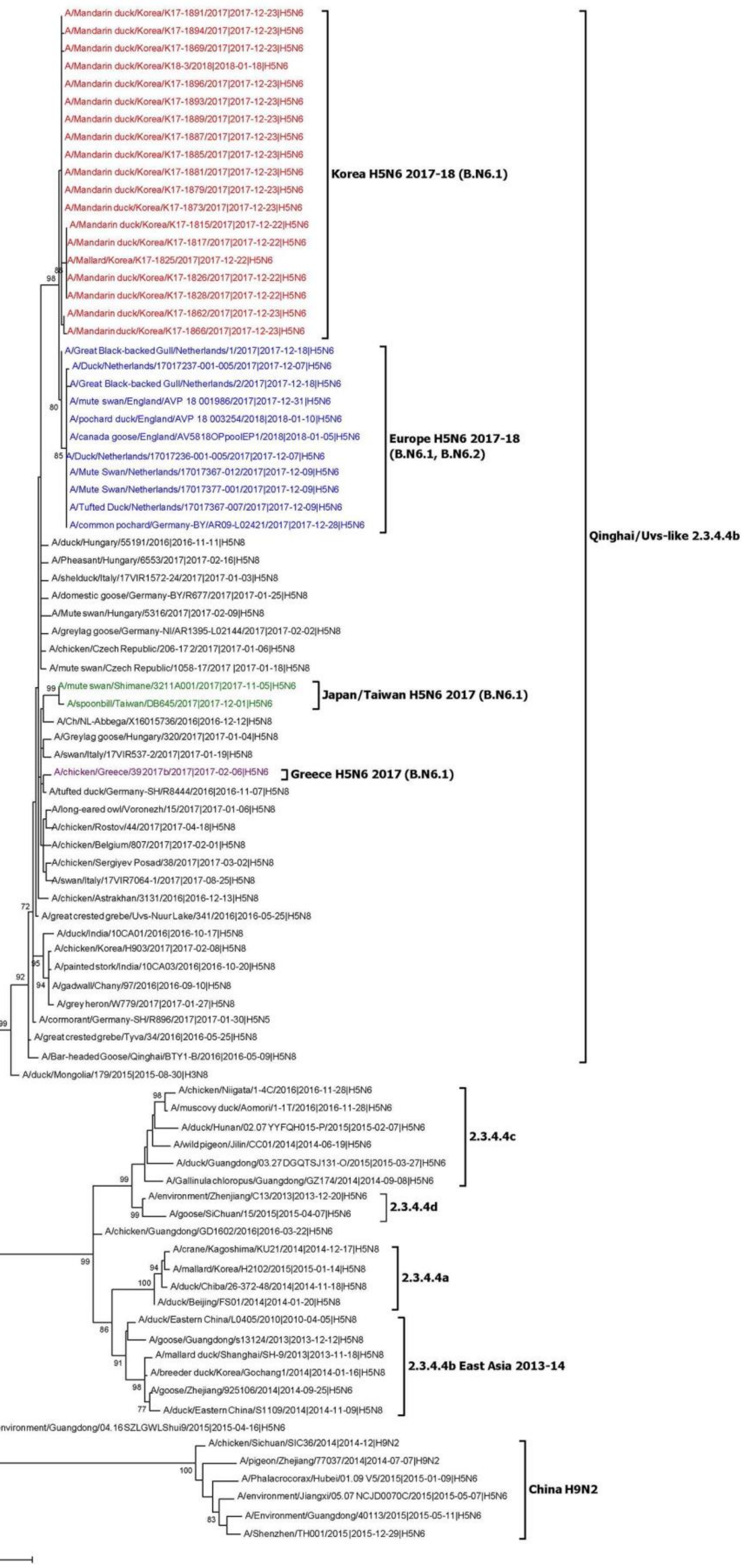
A/Mandarin duck/Korea/K17-1891/2017|2017-12-23|H5N6
A/Mandarin duck/Korea/K17-1894/2017|2017-12-23|H5N6
A/Mandarin duck/Korea/K17-1869/2017|2017-12-23|H5N6
A/Mandarin duck/Korea/K18-3/2018|2018-01-18|H5N6
A/Mandarin duck/Korea/K17-1896/2017|2017-12-23|H5N6
A/Mandarin duck/Korea/K17-1893/2017|2017-12-23|H5N6
A/Mandarin duck/Korea/K17-1889/2017|2017-12-23|H5N6
A/Mandarin duck/Korea/K17-1887/2017|2017-12-23|H5N6
A/Mandarin duck/Korea/K17-1885/2017|2017-12-23|H5N6
A/Mandarin duck/Korea/K17-1881/2017|2017-12-23|H5N6
A/Mandarin duck/Korea/K17-1879/2017|2017-12-23|H5N6
A/Mandarin duck/Korea/K17-1873/2017|2017-12-23|H5N6
A/Mandarin duck/Korea/K17-1815/2017|2017-12-22|H5N6
A/Mandarin duck/Korea/K17-1817/2017|2017-12-22|H5N6
A/Mallard/Korea/K17-1825/2017|2017-12-22|H5N6
A/Mandarin duck/Korea/K17-1826/2017|2017-12-22|H5N6
A/Mandarin duck/Korea/K17-1828/2017|2017-12-22|H5N6
A/Mandarin duck/Korea/K17-1862/2017|2017-12-23|H5N6
A/Mandarin duck/Korea/K17-1866/2017|2017-12-23|H5N6
Korea H5N6 2017-18 (B.N6.1)
98
A/Great Black-backed Gull/Netherlands/1/2017|2017-12-18|H5N6
A/Duck/Netherlands/17017237-001-005/2017|2017-12-07|H5N6
A/Great Black-backed Gull/Netherlands/2/2017|2017-12-18|H5N6
A/mute swan/England/AVP 18 001986/2017|2017-12-31|H5N6
A/pochard duck/England/AVP 18 003254/2018|2018-01-10|H5N6
A/canada goose/England/AV5818OPpoolEP1/2018|2018-01-05|H5N6
A/Duck/Netherlands/17017236-001-005/2017|2017-12-07|H5N6
A/Mute Swan/Netherlands/17017367-012/2017|2017-12-09|H5N6
A/Mute Swan/Netherlands/17017377-001/2017|2017-12-09|H5N6
A/Tufted Duck/Netherlands/17017367-007/2017|2017-12-09|H5N6
A/common pochard/Germany-BY/AR09-L02421/2017|2017-12-28|H5N6
80
85
Europe H5N6 2017-18
(B.N6.1, B.N6.2)
A/duck/Hungary/55191/2016|2016-11-11|H5N8
A/Pheasant/Hungary/6553/2017|2017-02-16|H5N8
A/shelduck/Italy/17VIR1572-24/2017|2017-01-03|H5N8
A/domestic goose/Germany-BY/R677/2017|2017-01-25|H5N8
A/Mute swan/Hungary/5316/2017|2017-02-09|H5N8
A/greylag goose/Germany-NI/AR1395-L02144/2017|2017-02-02|H5N8
A/chicken/Czech Republic/206-17 2/2017|2017-01-06|H5N8
A/mute swan/Czech Republic/1058-17/2017 |2017-01-18|H5N8
99
A/mute swan/Shimane/3211A001/2017|2017-11-05|H5N6
A/spoonbill/Taiwan/DB645/2017|2017-12-01|H5N6
Japan/Taiwan H5N6 2017 (B.N6.1)
A/Ch/NL-Abbega/X16015736/2016|2016-12-12|H5N8
A/Greylag goose/Hungary/320/2017|2017-01-04|H5N8
A/swan/Italy/17VIR537-2/2017|2017-01-19|H5N8
A/chicken/Greece/39 2017b/2017|2017-02-06|H5N6
Greece H5N6 2017 (B.N6.1)
A/tufted duck/Germany-SH/R8444/2016|2016-11-07|H5N8
A/long-eared owl/Voronezh/15/2017|2017-01-06|H5N8
A/chicken/Rostov/44/2017|2017-04-18|H5N8
A/chicken/Belgium/807/2017|2017-02-01|H5N8
A/chicken/Sergiyev Posad/38/2017|2017-03-02|H5N8
A/swan/Italy/17VIR7064-1/2017|2017-08-25|H5N8
A/chicken/Astrakhan/3131/2016|2016-12-13|H5N8
72
A/great crested grebe/Uvs-Nuur Lake/341/2016|2016-05-25|H5N8
A/duck/India/10CA01/2016|2016-10-17|H5N8
A/chicken/Korea/H903/2017|2017-02-08|H5N8
95
A/painted stork/India/10CA03/2016|2016-10-20|H5N8
92
94
A/gadwall/Chany/97/2016|2016-09-10|H5N8
A/grey heron/W779/2017|2017-01-27|H5N8
99
A/cormorant/Germany-SH/R896/2017|2017-01-30|H5N5
A/great crested grebe/Tyva/34/2016|2016-05-25|H5N8
A/Bar-headed Goose/Qinghai/BTY1-B/2016|2016-05-09|H5N8
Qinghai/Uvs-like 2.3.4.4b
A/duck/Mongolia/179/2015|2015-08-30|H3N8
98
A/chicken/Niigata/1-4C/2016|2016-11-28|H5N6
A/muscovy duck/Aomori/1-1T/2016|2016-11-28|H5N6
A/duck/Hunan/02.07 YYFQH015-P/2015|2015-02-07|H5N6
A/wild pigeon/Jilin/CC01/2014|2014-06-19|H5N6
A/duck/Guangdong/03.27 DGQTSJ131-O/2015|2015-03-27|H5N6
99
A/Gallinula chloropus/Guangdong/GZ174/2014|2014-09-08|H5N6
2.3.4.4c
A/environment/Zhenjiang/C13/2013|2013-12-20|H5N6
99
A/goose/SiChuan/15/2015|2015-04-07|H5N6
2.3.4.4d
A/chicken/Guangdong/GD1602/2016|2016-03-22|H5N6
A/crane/Kagoshima/KU21/2014|2014-12-17|H5N8
99
94
A/mallard/Korea/H2102/2015|2015-01-14|H5N8
100
A/duck/Chiba/26-372-48/2014|2014-11-18|H5N8
A/duck/Beijing/FS01/2014|2014-01-20|H5N8
2.3.4.4a
A/duck/Eastern China/L0405/2010|2010-04-05|H5N8
86
A/goose/Guangdong/s13124/2013|2013-12-12|H5N8
A/mallard duck/Shanghai/SH-9/2013|2013-11-18|H5N8
91
A/breeder duck/Korea/Gochang1/2014|2014-01-16|H5N8
98
A/goose/Zhejiang/925106/2014|2014-09-25|H5N6
77
A/duck/Eastern China/S1109/2014|2014-11-09|H5N8
2.3.4.4b East Asia 2013-14
A/environment/Guangdong/04.16SZLGWLShui9/2015|2015-04-16|H5N6
A/chicken/Sichuan/SIC36/2014|2014-12|H9N2
A/pigeon/Zhejiang/77037/2014|2014-07-07|H9N2
100
A/Phalacrocorax/Hubei/01.09 V5/2015|2015-01-09|H5N6
A/environment/Jiangxi/05.07 NCJD0070C/2015|2015-05-07|H5N6
83
A/Environment/Guangdong/40113/2015|2015-05-11|H5N6
A/Shenzhen/TH001/2015|2015-12-29|H5N6
China H9N2

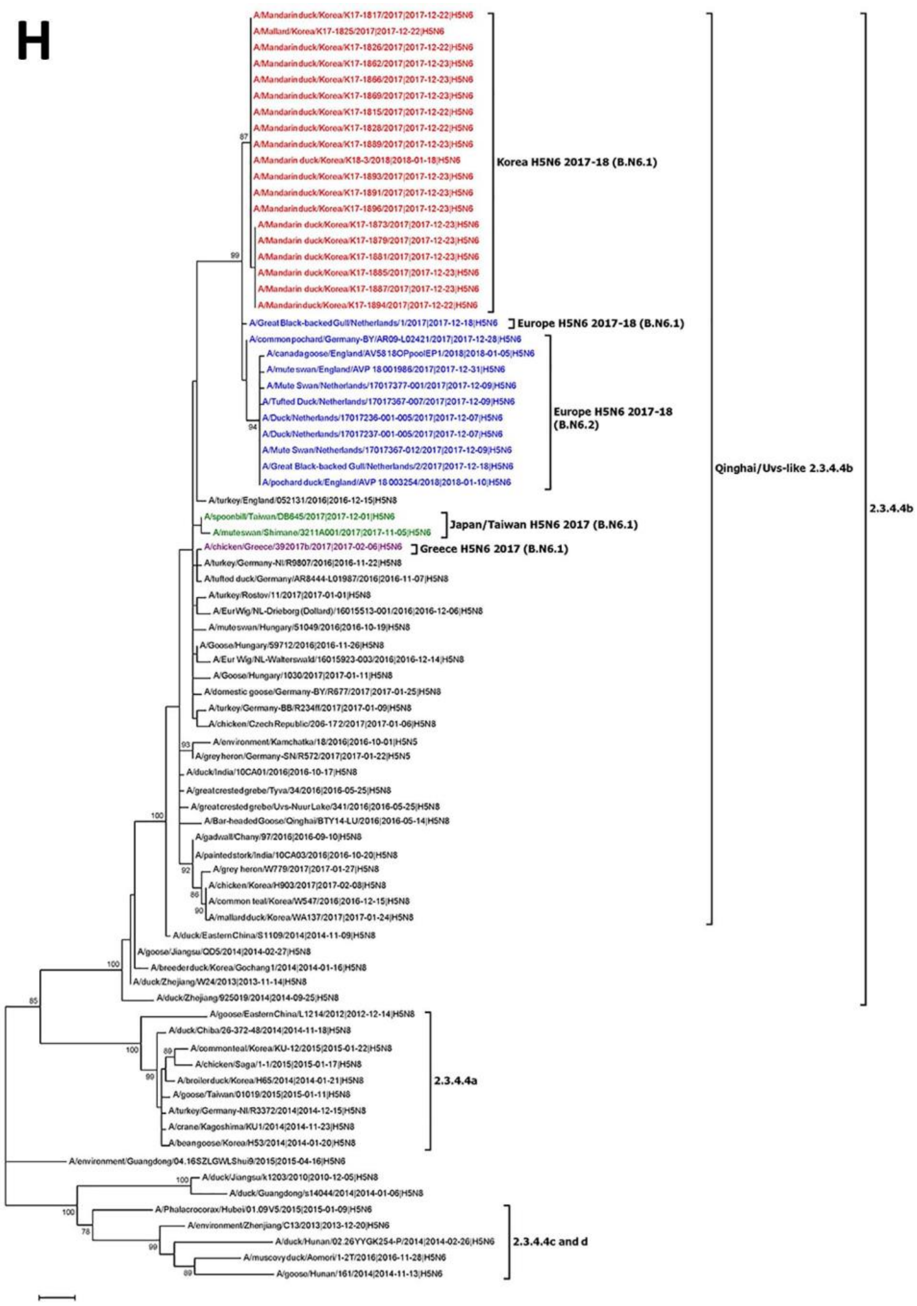

Technical Appendix 1 Figure 2. Maximum-likelihood phylogenetic tree of the PB2, PB1, PA, HA, NP, NA, M, and NS genes. The new subgroup B H5N6 isolates are colored according to the isolated location (South Korea, red; Europe, blue; Japan/Taiwan, green; Greece, purple). The percentages of replicate trees (>70%) in which the associated taxa clustered together in the bootstrap test (1,000 replicates) are shown next to the branches. Each cluster is labeled according to classification of Pohlmann et al. (*5*) and Beerens et al. (*6*).HA, hemagglutinin; M, matrix; NA, neuraminidase; NP, nucleoprotein; NS, nonstructural; PA, polymerase acidic; PB, polymerase basic. Scale bars indicate nucleotide substitutions per site.

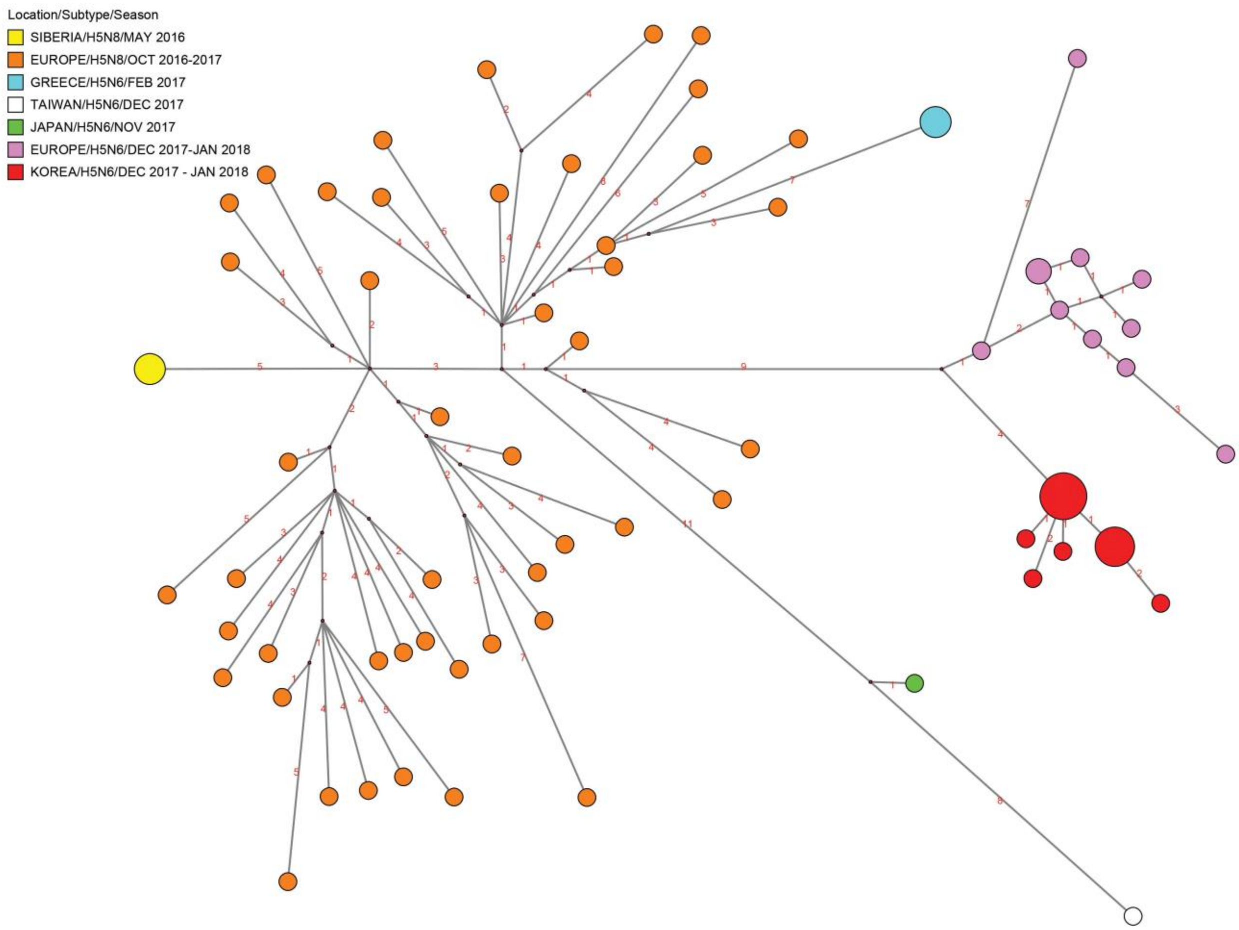

Technical Appendix 1 Figure 3. Median-joining phylogenetic network of subgroup B H5 highly pathogenic avian influenza viruses. The median-joining network was constructed from the HA gene. It includes all the most parsimonious trees linking the sequences. Each unique sequence is represented by a circle sized relative to its frequency in the dataset. Branch length is proportional to the number of mutations. Isolates are colored according to the location. HA, hemagglutinin.

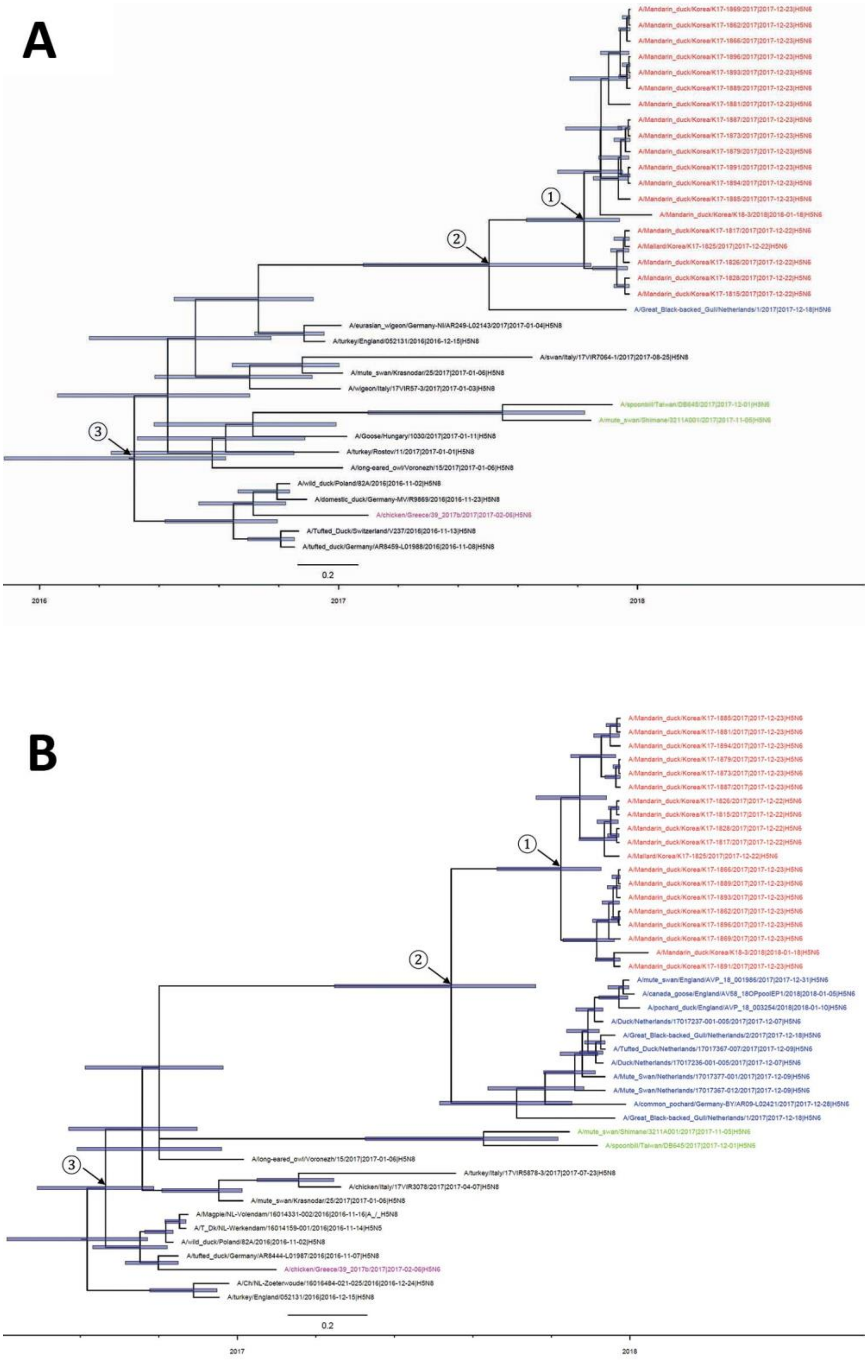
A
A/Mandarin_duck/Korea/K17-1869/2017|2017-12-23|H5N6
A/Mandarin_duck/Korea/K17-1862/2017|2017-12-23|H5N6
A/Mandarin_duck/Korea/K17-1866/2017|2017-12-23|H5N6
A/Mandarin_duck/Korea/K17-1896/2017|2017-12-23|H5N6
A/Mandarin_duck/Korea/K17-1893/2017|2017-12-23|H5N6
A/Mandarin_duck/Korea/K17-1889/2017|2017-12-23|H5N6
A/Mandarin_duck/Korea/K17-1881/2017|2017-12-23|H5N6
A/Mandarin_duck/Korea/K17-1887/2017|2017-12-23|H5N6
A/Mandarin_duck/Korea/K17-1873/2017|2017-12-23|H5N6
A/Mandarin_duck/Korea/K17-1879/2017|2017-12-23|H5N6
A/Mandarin_duck/Korea/K17-1891/2017|2017-12-23|H5N6
A/Mandarin_duck/Korea/K17-1894/2017|2017-12-23|H5N6
A/Mandarin_duck/Korea/K17-1885/2017|2017-12-23|H5N6
A/Mandarin_duck/Korea/K18-3/2018|2018-01-18|H5N6
A/Mandarin_duck/Korea/K17-1817/2017|2017-12-22|H5N6
A/Mallard/Korea/K17-1825/2017|2017-12-22|H5N6
A/Mandarin_duck/Korea/K17-1826/2017|2017-12-22|H5N6
A/Mandarin_duck/Korea/K17-1828/2017|2017-12-22|H5N6
A/Mandarin_duck/Korea/K17-1815/2017|2017-12-22|H5N6
A/Great_Black-backed_Gull/Netherlands/1/2017|2017-12-18|H5N6
A/eurasian_wigeon/Germany-NI/AR249-L02143/2017|2017-01-04|H5N8
A/turkey/England/052131/2016|2016-12-15|H5N8
A/swan/Italy/17VIR7064-1/2017|2017-08-25|H5N8
A/mute_swan/Krasnodar/25/2017|2017-01-06|H5N8
A/wigeon/Italy/17VIR57-3/2017|2017-01-03|H5N8
A/spoonbill/Taiwan/DB645/2017|2017-12-01|H5N6
A/mute_swan/Shimane/3211A001/2017|2017-11-05|H5N6
A/Goose/Hungary/1030/2017|2017-01-11|H5N8
A/turkey/Rostov/11/2017|2017-01-01|H5N8
A/long-eared_owl/Voronezh/15/2017|2017-01-06|H5N8
A/wild_duck/Poland/82A/2016|2016-11-02|H5N8
A/domestic_duck/Germany-MV/R9869/2016|2016-11-23|H5N8
A/chicken/Greece/39_2017b/2017|2017-02-06|H5N6
A/Tufted_Duck/Switzerland/V237/2016|2016-11-13|H5N8
A/tufted_duck/Germany/AR8459-L01988/2016|2016-11-08|H5N8
0.2
2016
2017
2018
B
A/Mandarin_duck/Korea/K17-1885/2017|2017-12-23|H5N6
A/Mandarin_duck/Korea/K17-1881/2017|2017-12-23|H5N6
A/Mandarin_duck/Korea/K17-1894/2017|2017-12-23|H5N6
A/Mandarin_duck/Korea/K17-1879/2017|2017-12-23|H5N6
A/Mandarin_duck/Korea/K17-1873/2017|2017-12-23|H5N6
A/Mandarin_duck/Korea/K17-1887/2017|2017-12-23|H5N6
A/Mandarin_duck/Korea/K17-1826/2017|2017-12-22|H5N6
A/Mandarin_duck/Korea/K17-1815/2017|2017-12-22|H5N6
A/Mandarin_duck/Korea/K17-1828/2017|2017-12-22|H5N6
A/Mandarin_duck/Korea/K17-1817/2017|2017-12-22|H5N6
A/Mallard/Korea/K17-1825/2017|2017-12-22|H5N6
A/Mandarin_duck/Korea/K17-1866/2017|2017-12-23|H5N6
A/Mandarin_duck/Korea/K17-1889/2017|2017-12-23|H5N6
A/Mandarin_duck/Korea/K17-1893/2017|2017-12-23|H5N6
A/Mandarin_duck/Korea/K17-1862/2017|2017-12-23|H5N6
A/Mandarin_duck/Korea/K17-1896/2017|2017-12-23|H5N6
A/Mandarin_duck/Korea/K17-1869/2017|2017-12-23|H5N6
A/Mandarin_duck/Korea/K18-3/2018|2018-01-18|H5N6
A/Mandarin_duck/Korea/K17-1891/2017|2017-12-23|H5N6
A/mute_swan/England/AVP_18_001986/2017|2017-12-31|H5N6
A/canada_goose/England/AV58_18OPpoolEP1/2018|2018-01-05|H5N6
A/pochard_duck/England/AVP_18_003254/2018|2018-01-10|H5N6
A/Duck/Netherlands/17017237-001-005/2017|2017-12-07|H5N6
A/Great_Black-backed_Gull/Netherlands/2/2017|2017-12-18|H5N6
A/Tufted_Duck/Netherlands/17017367-007/2017|2017-12-09|H5N6
A/Duck/Netherlands/17017236-001-005/2017|2017-12-07|H5N6
A/Mute_Swan/Netherlands/17017377-001/2017|2017-12-09|H5N6
A/Mute_Swan/Netherlands/17017367-012/2017|2017-12-09|H5N6
A/common_pochard/Germany-BY/AR09-L02421/2017|2017-12-28|H5N6
A/Great_Black-backed_Gull/Netherlands/1/2017|2017-12-18|H5N6
A/mute_swan/Shimane/3211A001/2017|2017-11-05|H5N6
A/spoonbill/Taiwan/DB645/2017|2017-12-01|H5N6
A/long-eared_owl/Voronezh/15/2017|2017-01-06|H5N8
A/turkey/Italy/17VIR5878-3/2017|2017-07-23|H5N8
A/chicken/Italy/17VIR3078/2017|2017-04-07|H5N8
A/mute_swan/Krasnodar/25/2017|2017-01-06|H5N8
A/Magpie/NL-Volendam/16014331-002/2016|2016-11-16|A_/_H5N8
A/T_Dk/NL-Werkendam/16014159-001/2016|2016-11-14|H5N5
A/wild_duck/Poland/82A/2016|2016-11-02|H5N8
A/tufted_duck/Germany/AR8444-L01987/2016|2016-11-07|H5N8
A/chicken/Greece/39_2017b/2017|2017-02-06|H5N6
A/Ch/NL-Zoeterwoude/16016484-021-025/2016|2016-12-24|H5N8
A/turkey/England/052131/2016|2016-12-15|H5N8
0.2
2017
2018

C

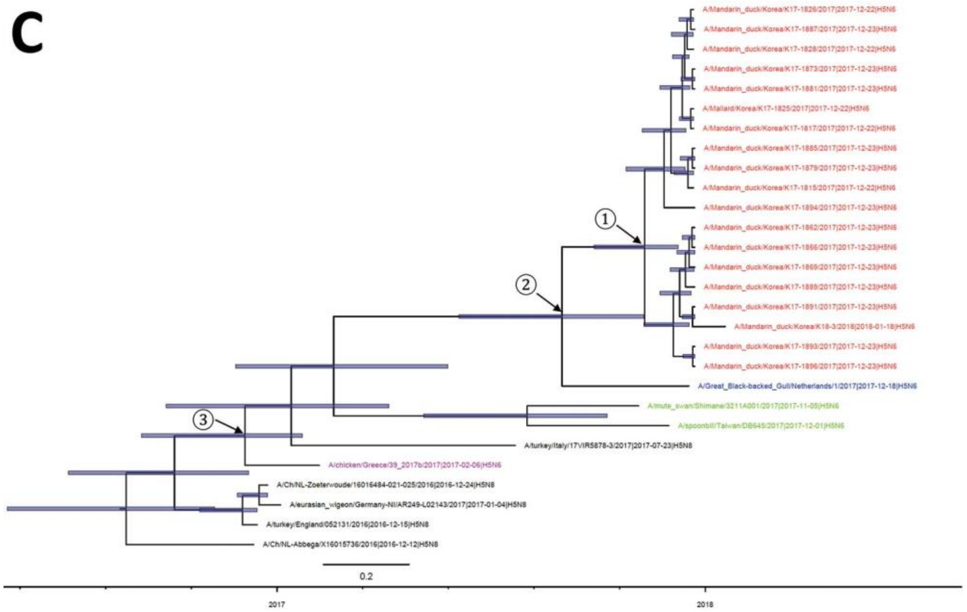
A/Mandarin_duck/Korea/K17-1826/2017|2017-12-22|H5N6
A/Mandarin_duck/Korea/K17-1887/2017|2017-12-23|H5N6
A/Mandarin_duck/Korea/K17-1828/2017|2017-12-22|H5N6
A/Mandarin_duck/Korea/K17-1873/2017|2017-12-23|H5N6
A/Mandarin_duck/Korea/K17-1881/2017|2017-12-23|H5N6
A/Mallard/Korea/K17-1825/2017|2017-12-22|H5N6
A/Mandarin_duck/Korea/K17-1817/2017|2017-12-22|H5N6
A/Mandarin_duck/Korea/K17-1885/2017|2017-12-23|H5N6
A/Mandarin_duck/Korea/K17-1879/2017|2017-12-23|H5N6
A/Mandarin_duck/Korea/K17-1815/2017|2017-12-22|H5N6
A/Mandarin_duck/Korea/K17-1894/2017|2017-12-23|H5N6
①
A/Mandarin_duck/Korea/K17-1862/2017|2017-12-23|H5N6
A/Mandarin_duck/Korea/K17-1866/2017|2017-12-23|H5N6
A/Mandarin_duck/Korea/K17-1869/2017|2017-12-23|H5N6
②
A/Mandarin_duck/Korea/K17-1889/2017|2017-12-23|H5N6
A/Mandarin_duck/Korea/K17-1891/2017|2017-12-23|H5N6
A/Mandarin_duck/Korea/K18-3/2018|2018-01-18|H5N6
A/Mandarin_duck/Korea/K17-1893/2017|2017-12-23|H5N6
A/Mandarin_duck/Korea/K17-1896/2017|2017-12-23|H5N6
A/Great_Black-backed_Gull/Netherlands/1/2017|2017-12-18|H5N6
A/mute_swan/Shimane/3211A001/2017|2017-11-05|H5N6
A/spoonbill/Taiwan/DB645/2017|2017-12-01|H5N6
③
A/turkey/Italy/17VIR5878-3/2017|2017-07-23|H5N8
A/chicken/Greece/39_2017b/2017|2017-02-06|H5N6
A/Ch/NL-Zoeterwoude/16016484-021-025/2016|2016-12-24|H5N8
A/eurasian_wigeon/Germany-NI/AR249-L02143/2017|2017-01-04|H5N8
A/turkey/England/052131/2016|2016-12-15|H5N8
A/Ch/NL-Abbega/X16015736/2016|2016-12-12|H5N8
0.2
2017
2018

D

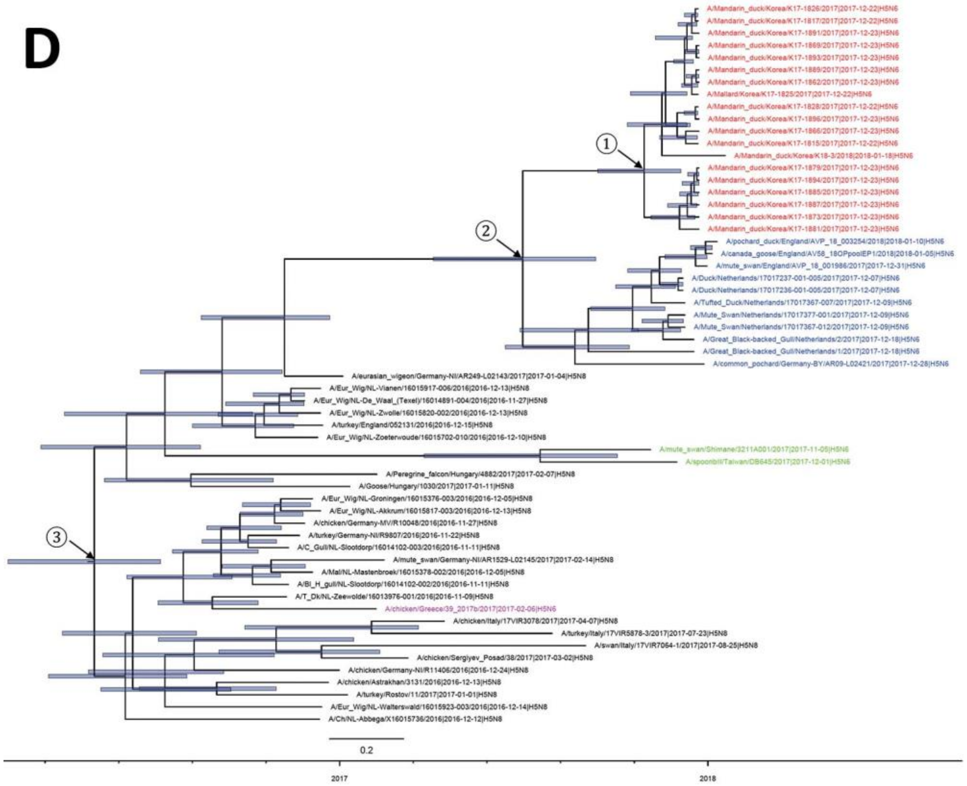
A/Mandarin_duck/Korea/K17-1826/2017|2017-12-22|H5N6
A/Mandarin_duck/Korea/K17-1817/2017|2017-12-22|H5N6
A/Mandarin_duck/Korea/K17-1891/2017|2017-12-23|H5N6
A/Mandarin_duck/Korea/K17-1869/2017|2017-12-23|H5N6
A/Mandarin_duck/Korea/K17-1893/2017|2017-12-23|H5N6
A/Mandarin_duck/Korea/K17-1889/2017|2017-12-23|H5N6
A/Mandarin_duck/Korea/K17-1862/2017|2017-12-23|H5N6
A/Mallard/Korea/K17-1825/2017|2017-12-22|H5N6
A/Mandarin_duck/Korea/K17-1828/2017|2017-12-22|H5N6
A/Mandarin_duck/Korea/K17-1896/2017|2017-12-23|H5N6
A/Mandarin_duck/Korea/K17-1866/2017|2017-12-23|H5N6
A/Mandarin_duck/Korea/K17-1815/2017|2017-12-22|H5N6
①
A/Mandarin_duck/Korea/K18-3/2018|2018-01-18|H5N6
A/Mandarin_duck/Korea/K17-1879/2017|2017-12-23|H5N6
A/Mandarin_duck/Korea/K17-1894/2017|2017-12-23|H5N6
A/Mandarin_duck/Korea/K17-1885/2017|2017-12-23|H5N6
A/Mandarin_duck/Korea/K17-1887/2017|2017-12-23|H5N6
A/Mandarin_duck/Korea/K17-1873/2017|2017-12-23|H5N6
A/Mandarin_duck/Korea/K17-1881/2017|2017-12-23|H5N6
②
A/pochard_duck/England/AVP_18_003254/2018|2018-01-10|H5N6
A/canada_goose/England/AV58_18OPpoolEP1/2018|2018-01-05|H5N6
A/mute_swan/England/AVP_18_001986/2017|2017-12-31|H5N6
A/Duck/Netherlands/17017237-001-005/2017|2017-12-07|H5N6
A/Duck/Netherlands/17017236-001-005/2017|2017-12-07|H5N6
A/Tufted_Duck/Netherlands/17017367-007/2017|2017-12-09|H5N6
A/Mute_Swan/Netherlands/17017377-001/2017|2017-12-09|H5N6
A/Mute_Swan/Netherlands/17017367-012/2017|2017-12-09|H5N6
A/Great_Black-backed_Gull/Netherlands/2/2017|2017-12-18|H5N6
A/Great_Black-backed_Gull/Netherlands/1/2017|2017-12-18|H5N6
A/common_pochard/Germany-BY/AR09-L02421/2017|2017-12-28|H5N6
A/eurasian_wigeon/Germany-NI/AR249-L02143/2017|2017-01-04|H5N8
A/Eur_Wig/NL-Vianen/16015917-006/2016|2016-12-13|H5N8
A/Eur_Wig/NL-De_Waal_(Texel)/16014891-004/2016|2016-11-27|H5N8
A/Eur_Wig/NL-Zwolle/16015820-002/2016|2016-12-13|H5N8
A/turkey/England/052131/2016|2016-12-15|H5N8
A/Eur_Wig/NL-Zoeterwoude/16015702-010/2016|2016-12-10|H5N8
A/mute_swan/Shimane/3211A001/2017|2017-11-05|H5N6
A/spoonbill/Taiwan/DB645/2017|2017-12-01|H5N6
A/Peregrine_falcon/Hungary/4882/2017|2017-02-07|H5N8
A/Goose/Hungary/1030/2017|2017-01-11|H5N8
A/Eur_Wig/NL-Groningen/16015376-003/2016|2016-12-05|H5N8
A/Eur_Wig/NL-Akkrum/16015817-003/2016|2016-12-13|H5N8
A/chicken/Germany-MV/R10048/2016|2016-11-27|H5N8
A/turkey/Germany-NI/R9807/2016|2016-11-22|H5N8
③
A/C_Gull/NL-Slootdorp/16014102-003/2016|2016-11-11|H5N8
A/mute_swan/Germany-NI/AR1529-L02145/2017|2017-02-14|H5N8
A/Mal/NL-Mastenbroek/16015378-002/2016|2016-12-05|H5N8
A/Bl_H_gull/NL-Slootdorp/16014102-002/2016|2016-11-11|H5N8
A/T_Dk/NL-Zeewolde/16013976-001/2016|2016-11-09|H5N8
A/chicken/Greece/39_2017b/2017|2017-02-06|H5N6
A/chicken/Italy/17VIR3078/2017|2017-04-07|H5N8
A/turkey/Italy/17VIR5878-3/2017|2017-07-23|H5N8
A/swan/Italy/17VIR7064-1/2017|2017-08-25|H5N8
A/chicken/Sergiyev_Posad/38/2017|2017-03-02|H5N8
A/chicken/Germany-NI/R11406/2016|2016-12-24|H5N8
A/chicken/Astrakhan/3131/2016|2016-12-13|H5N8
A/turkey/Rostov/11/2017|2017-01-01|H5N8
A/Eur_Wig/NL-Walterswald/16015923-003/2016|2016-12-14|H5N8
A/Ch/NL-Abbega/X16015736/2016|2016-12-12|H5N8
0.2
2017
2018

E

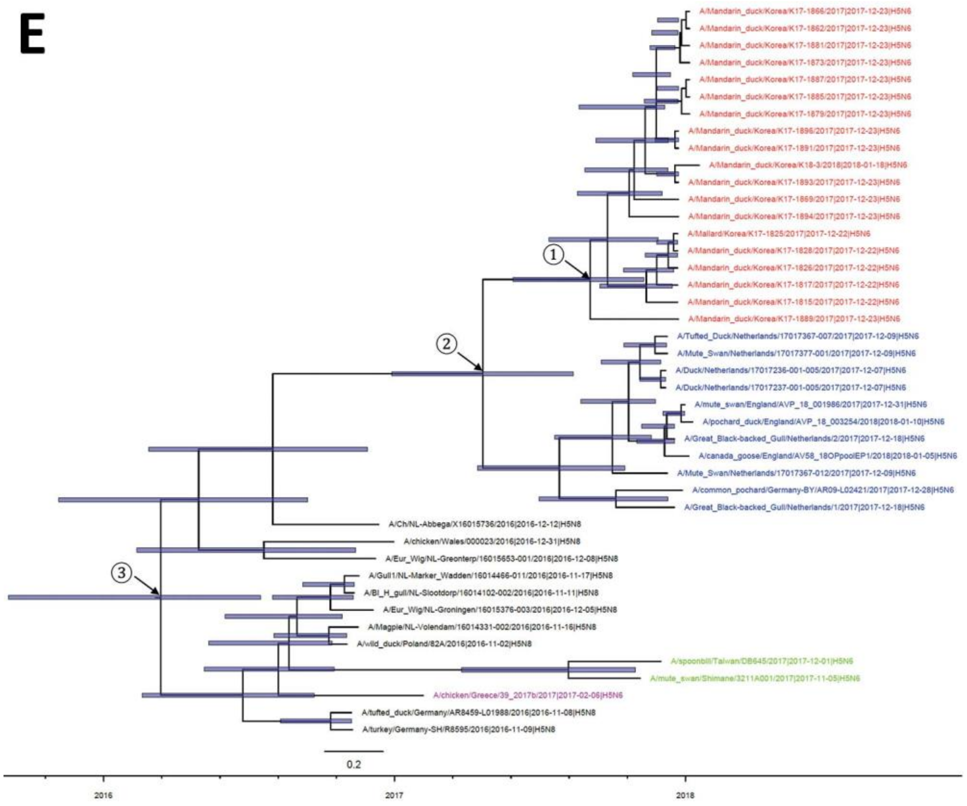

A/Mandarin_duck/Korea/K17-1866/2017|2017-12-23|H5N6
A/Mandarin_duck/Korea/K17-1862/2017|2017-12-23|H5N6
A/Mandarin_duck/Korea/K17-1881/2017|2017-12-23|H5N6
A/Mandarin_duck/Korea/K17-1873/2017|2017-12-23|H5N6
A/Mandarin_duck/Korea/K17-1887/2017|2017-12-23|H5N6
A/Mandarin_duck/Korea/K17-1885/2017|2017-12-23|H5N6
A/Mandarin_duck/Korea/K17-1879/2017|2017-12-23|H5N6
A/Mandarin_duck/Korea/K17-1896/2017|2017-12-23|H5N6
A/Mandarin_duck/Korea/K17-1891/2017|2017-12-23|H5N6
A/Mandarin_duck/Korea/K18-3/2018|2018-01-18|H5N6
A/Mandarin_duck/Korea/K17-1893/2017|2017-12-23|H5N6
A/Mandarin_duck/Korea/K17-1869/2017|2017-12-23|H5N6
A/Mandarin_duck/Korea/K17-1894/2017|2017-12-23|H5N6
A/Mallard/Korea/K17-1825/2017|2017-12-22|H5N6
A/Mandarin_duck/Korea/K17-1828/2017|2017-12-22|H5N6
A/Mandarin_duck/Korea/K17-1826/2017|2017-12-22|H5N6
A/Mandarin_duck/Korea/K17-1817/2017|2017-12-22|H5N6
A/Mandarin_duck/Korea/K17-1815/2017|2017-12-22|H5N6
A/Mandarin_duck/Korea/K17-1889/2017|2017-12-23|H5N6
A/Tufted_Duck/Netherlands/17017367-007/2017|2017-12-09|H5N6
A/Mute_Swan/Netherlands/17017377-001/2017|2017-12-09|H5N6
A/Duck/Netherlands/17017236-001-005/2017|2017-12-07|H5N6
A/Duck/Netherlands/17017237-001-005/2017|2017-12-07|H5N6
A/mute_swan/England/AVP_18_001986/2017|2017-12-31|H5N6
A/pochard_duck/England/AVP_18_003254/2018|2018-01-10|H5N6
A/Great_Black-backed_Gull/Netherlands/2/2017|2017-12-18|H5N6
A/canada_goose/England/AV58_18OPpoolEP1/2018|2018-01-05|H5N6
A/Mute_Swan/Netherlands/17017367-012/2017|2017-12-09|H5N6
A/common_pochard/Germany-BY/AR09-L02421/2017|2017-12-28|H5N6
A/Great_Black-backed_Gull/Netherlands/1/2017|2017-12-18|H5N6
A/Ch/NL-Abbega/X16015736/2016|2016-12-12|H5N8
A/chicken/Wales/000023/2016|2016-12-31|H5N8
A/Eur_Wig/NL-Greonterp/16015653-001/2016|2016-12-08|H5N8
A/Gull1/NL-Marker_Wadden/16014466-011/2016|2016-11-17|H5N8
A/Bl_H_gull/NL-Slootdorp/16014102-002/2016|2016-11-11|H5N8
A/Eur_Wig/NL-Groningen/16015376-003/2016|2016-12-05|H5N8
A/Magpie/NL-Volendam/16014331-002/2016|2016-11-16|H5N8
A/wild_duck/Poland/82A/2016|2016-11-02|H5N8
A/spoonbill/Taiwan/DB645/2017|2017-12-01|H5N6
A/mute_swan/Shimane/3211A001/2017|2017-11-05|H5N6
A/chicken/Greece/39_2017b/2017|2017-02-06|H5N6
A/tufted_duck/Germany/AR8459-L01988/2016|2016-11-08|H5N8
A/turkey/Germany-SH/R8595/2016|2016-11-09|H5N8
①
②
③
0.2
2016
2017
2018

F

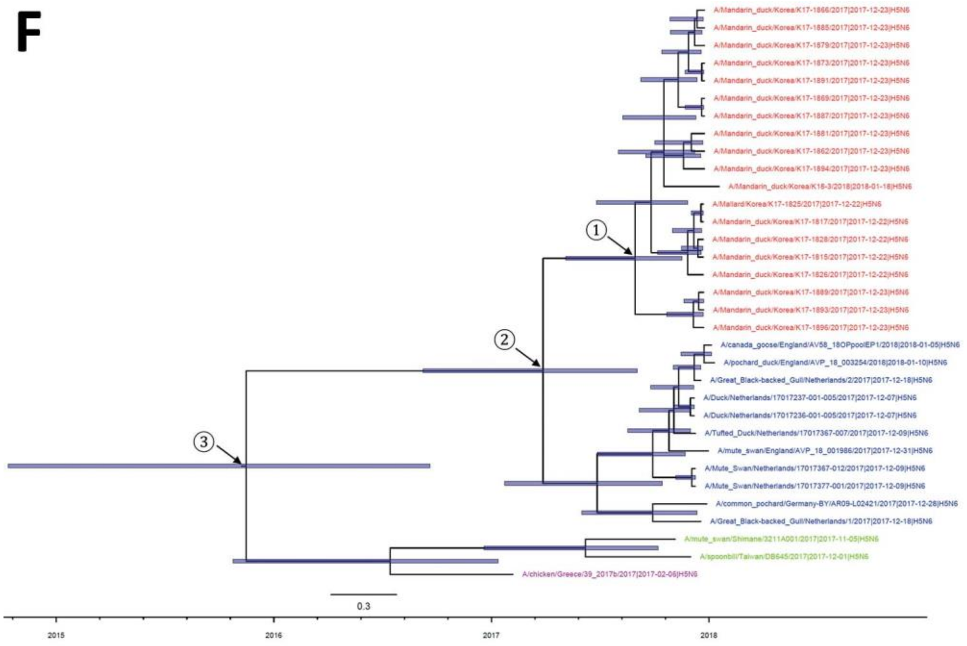

A/Mandarin_duck/Korea/K17-1866/2017|2017-12-23|H5N6
A/Mandarin_duck/Korea/K17-1885/2017|2017-12-23|H5N6
A/Mandarin_duck/Korea/K17-1879/2017|2017-12-23|H5N6
A/Mandarin_duck/Korea/K17-1873/2017|2017-12-23|H5N6
A/Mandarin_duck/Korea/K17-1891/2017|2017-12-23|H5N6
A/Mandarin_duck/Korea/K17-1869/2017|2017-12-23|H5N6
A/Mandarin_duck/Korea/K17-1887/2017|2017-12-23|H5N6
A/Mandarin_duck/Korea/K17-1881/2017|2017-12-23|H5N6
A/Mandarin_duck/Korea/K17-1862/2017|2017-12-23|H5N6
A/Mandarin_duck/Korea/K17-1894/2017|2017-12-23|H5N6
A/Mandarin_duck/Korea/K18-3/2018|2018-01-18|H5N6
A/Mallard/Korea/K17-1825/2017|2017-12-22|H5N6
A/Mandarin_duck/Korea/K17-1817/2017|2017-12-22|H5N6
A/Mandarin_duck/Korea/K17-1828/2017|2017-12-22|H5N6
A/Mandarin_duck/Korea/K17-1815/2017|2017-12-22|H5N6
A/Mandarin_duck/Korea/K17-1826/2017|2017-12-22|H5N6
A/Mandarin_duck/Korea/K17-1889/2017|2017-12-23|H5N6
A/Mandarin_duck/Korea/K17-1893/2017|2017-12-23|H5N6
A/Mandarin_duck/Korea/K17-1896/2017|2017-12-23|H5N6
A/canada_goose/England/AV58_18OPpoolEP1/2018|2018-01-05|H5N6
A/pochard_duck/England/AVP_18_003254/2018|2018-01-10|H5N6
A/Great_Black-backed_Gull/Netherlands/2/2017|2017-12-18|H5N6
A/Duck/Netherlands/17017237-001-005/2017|2017-12-07|H5N6
A/Duck/Netherlands/17017236-001-005/2017|2017-12-07|H5N6
A/Tufted_Duck/Netherlands/17017367-007/2017|2017-12-09|H5N6
A/mute_swan/England/AVP_18_001986/2017|2017-12-31|H5N6
A/Mute_Swan/Netherlands/17017367-012/2017|2017-12-09|H5N6
A/Mute_Swan/Netherlands/17017377-001/2017|2017-12-09|H5N6
A/common_pochard/Germany-BY/AR09-L02421/2017|2017-12-28|H5N6
A/Great_Black-backed_Gull/Netherlands/1/2017|2017-12-18|H5N6
A/mute_swan/Shimane/3211A001/2017|2017-11-05|H5N6
A/spoonbill/Taiwan/DB645/2017|2017-12-01|H5N6
A/chicken/Greece/39_2017b/2017|2017-02-06|H5N6
①
②
③
0.3
2015
2016
2017
2018

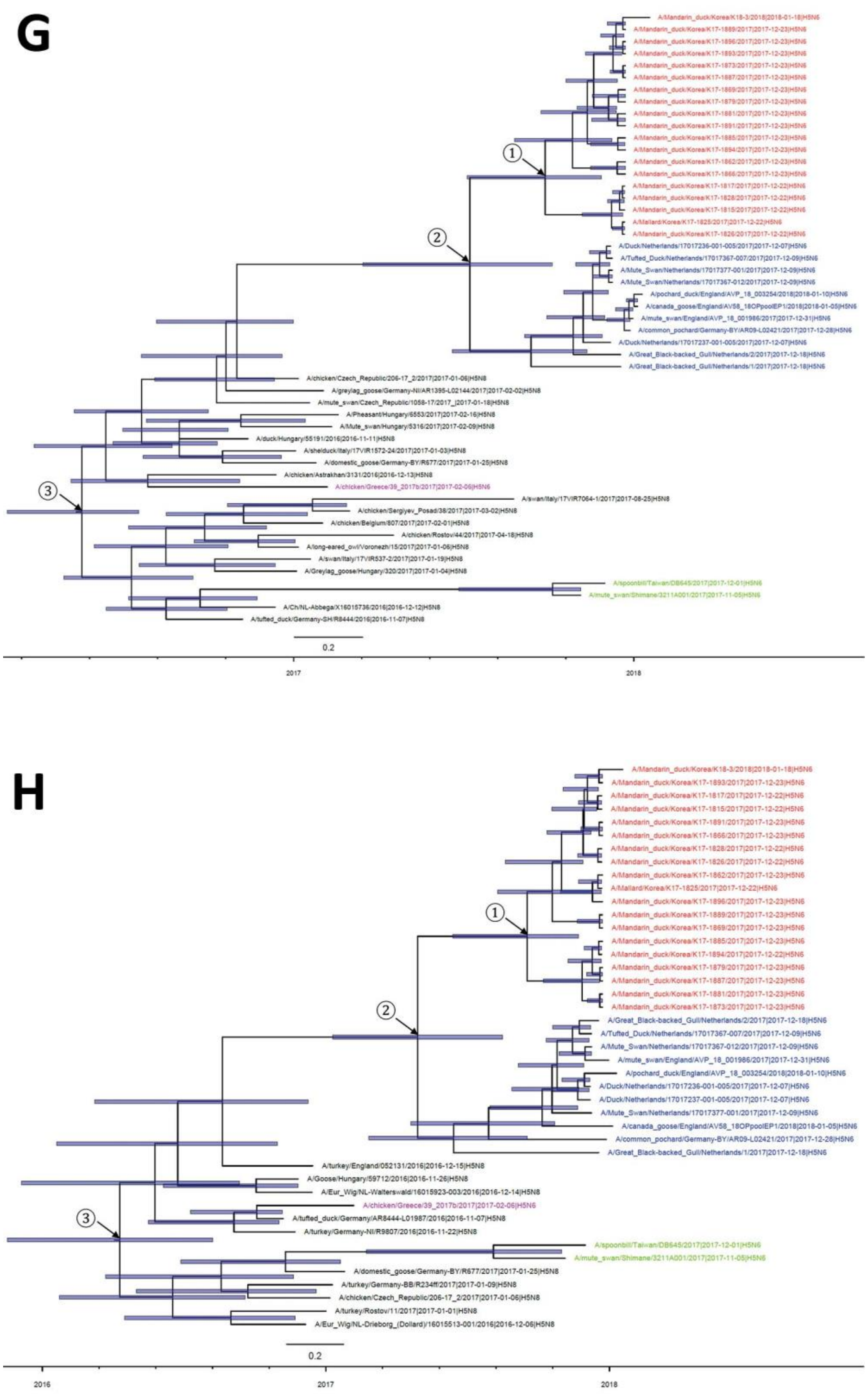

Technical Appendix 1 Figure 4. Temporally structured maximum clade credibility phylogenetic tree (years on the horizontal axis) of the PB2, PB1, PA, HA, NP, NA, M, and NS genes of subgroup B HPAIV. The new subgroup B H5N6 Isolates are colored according to the isolated location (South Korea, red;

Europe, blue; Japan/Taiwan, green; and Greece, purple). The horizontal bars indicate the 95% highest posterior density (HPD) intervals of the most recent common ancestor. HA, hemagglutinin; HPD, highest posterior density; M, matrix; NA, neuraminidase; NP, nucleoprotein; NS, nonstructural; PA, polymerase acidic; PB, polymerase basic. Scale bars indicate nucleotide substitutions per site.
